# Machine Learning-based Models for Outpatient Prescription of Kampo Formulations: An Analysis of a Health Insurance Claims Database

**DOI:** 10.2188/jea.JE20220089

**Published:** 2024-01-05

**Authors:** Hayato Yamana, Akira Okada, Sachiko Ono, Nobuaki Michihata, Taisuke Jo, Hideo Yasunaga

**Affiliations:** 1 Department of Health Services Research, Graduate School of Medicine, The University of Tokyo, Tokyo, Japan; 2 Department of Prevention of Diabetes and Lifestyle-Related Diseases, Graduate School of Medicine, The University of Tokyo, Tokyo, Japan; 3 Department of Eat-loss Medicine, Graduate School of Medicine, The University of Tokyo, Tokyo, Japan; 4 Department of Clinical Epidemiology and Health Economics, School of Public Health, The University of Tokyo, Tokyo, Japan

**Keywords:** administrative database, Kampo medicine, machine learning

## Abstract

**Background:**

Despite the widespread practice of Japanese traditional Kampo medicine, the characteristics of patients receiving various Kampo formulations have not been documented in detail. We applied a machine learning model to a health insurance claims database to identify the factors associated with the use of Kampo formulations.

**Methods:**

A 10% sample of enrollees of the JMDC Claims Database in 2018 and 2019 was used to create the training and testing sets, respectively. Logistic regression analyses with lasso regularization were performed in the training set to construct models with prescriptions of 10 commonly used Kampo formulations in 1 year as the dependent variable and data of the preceding year as independent variables. Models were applied to the testing set to calculate the C-statistics. Additionally, the performance of simplified scores using 10 or 5 variables were evaluated.

**Results:**

There were 338,924 and 399,174 enrollees in the training and testing sets, respectively. The commonly prescribed Kampo formulations included kakkonto, bakumondoto, and shoseityuto. Based on the lasso models, the C-statistics ranged from 0.643 (maoto) to 0.888 (tokishakuyakusan). The models identified both the common determinants of different Kampo formulations and the specific characteristics associated with particular Kampo formulations. The simplified scores were slightly inferior to full models.

**Conclusion:**

Lasso regression models showed good performance for explaining various Kampo prescriptions from claims data. The models identified the characteristics associated with Kampo formulation use.

## INTRODUCTION

Kampo is a traditional Japanese medicine that uses formulae of natural agents.^1^ Clinical studies have reported the effectiveness of Kampo formulations for numerous conditions.^2^^–^^4^ In addition, large-scale observational studies using administrative databases have recently been conducted and complemented clinical trials. Among such real-world evidence are hangeshashinto as supportive therapy in chemotherapy, and daikenchuto for postoperative ileus, enteral feeding intolerance, and chronic obstructive pulmonary disease.^5^^–^^8^

According to survey studies, more than 80% of Japanese physicians use Kampo products in daily clinical practice.^9^^,^^10^ In a study using a health insurance claims database, 13.5% of enrollees received Kampo prescriptions within 1 year.^11^ However, despite the widespread use of Kampo products, the characteristics of patients receiving them have not been documented in detail. Previous studies using claims databases have thus far provided limited information because they only used diagnostic records to describe patient characteristics.^11^^,^^12^

Health insurance claims databases contain large information on patient diagnosis, medical services availed, and prescription. Regression models can be applied to such databases, elucidating the characteristics that differentiate between those who were prescribed Kampo formulations and those who were not, and identifying candidates for Kampo formulation use. In addition, machine learning models are increasingly utilized to create accurate models from large databases. In particular, lasso regression enables parsimonious models with easily interpretable and applicable results. This study aimed to create models to explain outpatient prescription of different Kampo formulations by applying lasso regression to a health insurance claims database.

## METHODS

### Data source

We conducted a retrospective study using the JMDC Claims Database (JMDC Inc., Tokyo, Japan), an anonymized database containing health insurance claims data provided by employer health insurance groups. Enrollee information included sex, year and month of birth, the period over which the data were obtained, and employment status (employee or dependent family member). Family members in the same household can be linked using family identification code. All claims data for medical services covered by health insurance were recorded in the database. Diagnoses were recorded based on the International Statistical Classification of Diseases and Related Health Problems, 10th revision (ICD-10) codes, and information on whether the diagnosis was suspected or confirmed was also recorded. Fees for medical services were recorded using the Japanese codes for reimbursement. Prescribed drugs were classified according to the Anatomical Therapeutic Chemical Classification System (ATC) by the World Health Organization.

### Participants

First, we identified enrollees aged 20–59 years as of April 2018 from the database. To ensure a 1-year period before the index date (April 2018) for baseline characteristics, we excluded those who joined after April 2017. A 10% random sample of this cohort was used as the training set for model construction. The sampling rate was determined to guarantee 10 outcome cases per independent variable, assuming that 0.5% of the participants received prescription of a Kampo formulation and that 100 variables were identified. The testing set for model validation was extracted in a similar manner, with April 2019 as the index date.

### Variables

The year preceding the index date (April 2017–March 2018 and April 2018–March 2019 for training and testing sets, respectively) was used as the baseline period to obtain enrollee characteristics, and the year following the index date was the used to summarize Kampo prescriptions. Five basic enrollee characteristics were extracted, including age, sex, employment status, number of family members in the same household, and cumulative medical cost during the baseline period (as an indicator of overall medical service utilization). We also summarized all data on diagnoses, medical services availed, and prescriptions during the baseline period and created dichotomous variables as follows. Confirmed diagnoses were categorized into 22 variables based on the ICD-10 chapters (eg, Chapter I, A00–B99; Chapter II, C00–D48).^13^ Medical services were categorized into 14 variables using the Japanese codes for reimbursement (eg, A for consultation and hospitalization, B for disease management fee). Prescription records were categorized into 93 variables using the first three digits of the ATC codes (eg, A01, A02). The variables are presented in eTable 1, eTable 2, and eTable 3.

Data during the year following the index date were summarized to identify enrollees who received at least one prescription of each Kampo formulation. In this study, we evaluated 10 types of Kampo formulation with the largest number of enrollees prescribed among the training set.

### Statistical analysis

We performed separate analyses for ten formulations. In each analysis, we first fitted a logistic regression model with lasso regularization in the training set to model the prescription at individual level. All 134 candidate variables described above were entered into the model. Five-fold cross-validation was performed to obtain the model that achieved the minimal cross-validation mean deviance. We then applied the obtained model to the testing set and estimated the predicted probability of receiving prescription. C-statistic was calculated to evaluate the discriminatory ability of the model.

For a comparative purpose, we tested the performance of conventional logistic regression models that used all 134 variables. DeLong’s test was conducted to comapre the C-statistics between the lasso models and conventional logisitc regression models. Additionally, we created two scores using 10 and 5 variables that showed large absolute values of standardized coefficient in the lasso model. The score was calculated by adding the products of 10 or 5 variables and their non-standardized coefficients. The C-statistic of the scores were also evaluated in the testing set. Finally, we conducted analyses limiting the enrollees to those who did not receive a prescription of each Kampo formulation during the baseline year. All statistical analyses were conducted using Stata SE version 17.0 (StataCorp, College Station, TX, USA).

### Ethical considerations

The study was approved by the Institutional Review Board of the Graduate School of Medicine, The University of Tokyo. The requirement for informed consent was waived owing to the analysis of anonymized data.

## RESULTS

**Figure 1.**
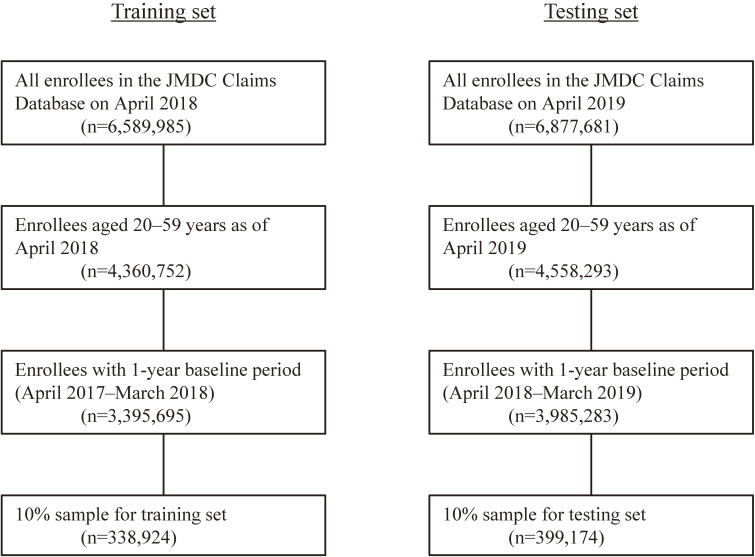
Selection of participants in the training and testing sets

Figure 1 shows a flowchart of the participant selection process. There were 338,924 and 399,174 enrollees included in the training and testing sets, respectively. Their basic characteristics are presented in Table 1. Those in the training and testing sets had similar characteristics. The 10 Kampo formulations that were prescribed at least once to most enrollees were kakkonto, bakumondoto, shoseiryuto, maoto, goreisan, kikyoto, maobushisaishinto, shakuyakukanzoto, tokishakuyakusan, and kakkontokasenkyushin’i. Overall, 15.5% (114,341/738,098) received at least one prescription of any Kampo formulation. The proportions of enrollees who were prescribed a Kampo formulation were 15.8% (*n* = 108,146) among 682,361 individuals followed for a full year and 11.1% (*n* = 6,195) among 55,737 individuals observed for shorter periods.

**Table 1.** Characteristics of enrollees in the training and testing sets

Characteristic	Training set (*n* = 338,924)	Testing set (*n* = 399,174)
Sex, *n* (%)		
Male	194,163 (57)	226,120 (57)
Female	144,761 (43)	173,054 (43)
Age, years		
mean (SD)	40.9 (10.8)	40.8 (10.9)
20–29, *n* (%)	63,361 (19)	76,228 (19)
30–39, *n* (%)	81,346 (24)	96,745 (24)
40–49, *n* (%)	107,259 (32)	123,379 (31)
50–59, *n* (%)	86,958 (26)	102,822 (26)
Employment status, *n* (%)		
Employee	245,890 (73)	295,983 (74)
Family member	93,034 (27)	103,191 (26)
Medical cost, yen, mean (SD)	119,188 (509,013)	117,176 (531,036)
Kampo formulation use, *n* (%)		
Kakkonto	7,980 (2.35)	9,143 (2.29)
Bakumondoto	6,241 (1.84)	7,081 (1.77)
Shoseiryuto	6,104 (1.80)	6,745 (1.69)
Maoto	4,434 (1.31)	4,710 (1.18)
Goreisan	3,298 (0.97)	3,923 (0.98)
Kikyoto	2,917 (0.86)	3,596 (0.90)
Maobushisaishinto	2,883 (0.85)	3,209 (0.80)
Shakuyakukanzoto	2,159 (0.64)	2,527 (0.63)
Tokishakuyakusan	2,062 (0.61)	2,489 (0.62)
Kakkontokasenkyushin’i	2,012 (0.59)	2,537 (0.64)

SD, standard deviation.

Table 2 summarizes the characteristics of the models for Kampo formulation use. The number of variables selected by applying the lasso models to the training set ranged from 49 (maoto) to 84 (shakuyakukanzoto). The lasso models had fair discriminatory abilities, with C-statistics in the testing set ranging from 0.643 (maoto) to 0.888 (tokishakuyakusan). DeLong’s test showed similar or better peformance of the lasso models compared with the conventional logistic regression models that used all 134 variables. The performance of the scores using 10 or 5 variables is also presented in Table 2. The C-statistics of the scores were smaller than those of the original lasso models; however, the differences between the three models were small. Table 3 presents the results of lasso regression analyses in enrollees without a prescription of each Kampo formulation during the baseline year. The C-statistics were lower than those of the main analysis.

**Table 2.** Characteristics of the models for Kampo formulation use

Kampo formulation	Lasso model				Conventional logisitc regression	*P*-value by DeLong’s test (lasso vs, conventional logisitc)	Score using 10 variables	Score using 5 variables
	Nonzero coefficients	Cross-validation mean deviance	Lambda (×10^−4^)	C-statistic (95% CI)	C-statistic (95% CI)		C-statistic (95% CI)	C-statistic (95% CI)
Kakkonto	81	0.207	2.47	0.720 (0.714–0.725)	0.720 (0.714–0.725)	0.894	0.711 (0.706–0.717)	0.706 (0.700–0.711)
Bakumondoto	78	0.173	2.11	0.704 (0.698–0.711)	0.704 (0.697–0.710)	0.017	0.699 (0.692–0.705)	0.694 (0.688–0.700)
Shoseiryuto	82	0.164	1.88	0.741 (0.735–0.748)	0.741 (0.735–0.747)	0.534	0.737 (0.730–0.743)	0.731 (0.725–0.738)
Maoto	49	0.136	2.37	0.643 (0.635–0.651)	0.642 (0.634–0.650)	0.225	0.638 (0.630–0.646)	0.624 (0.616–0.632)
Goreisan	61	0.101	1.98	0.732 (0.724–0.741)	0.730 (0.722–0.739)	0.003	0.725 (0.716–0.733)	0.716 (0.707–0.725)
Kikyoto	60	0.093	1.86	0.725 (0.717–0.734)	0.725 (0.716–0.733)	0.371	0.720 (0.711–0.729)	0.711 (0.702–0.720)
Maobushisaishinto	63	0.093	1.84	0.696 (0.687–0.706)	0.695 (0.685–0.705)	0.174	0.693 (0.683–0.702)	0.688 (0.678–0.698)
Shakuyakukanzoto	84	0.068	1.35	0.781 (0.771–0.791)	0.778 (0.768–0.788)	<0.001	0.778 (0.768–0.788)	0.770 (0.759–0.780)
Tokishakuyakusan	73	0.059	1.14	0.888 (0.883–0.894)	0.886 (0.881–0.892)	<0.001	0.886 (0.881–0.892)	0.883 (0.877–0.888)
Kakkontokasenkyushin’i	52	0.067	1.53	0.745 (0.734–0.756)	0.744 (0.733–0.754)	0.278	0.741 (0.730–0.751)	0.734 (0.724–0.745)

CI, confidence interval.

Nonzero coefficients, cross-validation mean deviance, and lambda were obtained from analyses of the training set (*n* = 338,924). C-statistics were calculated in the testing set (*n* = 399,174).

**Table 3.** Results of the lasso regression analyses in enrollees without prescription of each Kampo formulation during the baseline year

Kampo formulation	Training set		Testing set		Lasso model			
	Analyzed, *n*	Kampo use, *n* (%)	Analyzed, *n*	Kampo use, *n* (%)	Nonzero coefficients^a^	Cross-validation mean deviance^a^	Lambda (×10^−4^)^a^	C-statistic (95% confidence interval)^b^
Kakkonto	330,320	6,306 (1.91)	389,550	7,234 (1.86)	69	0.181	2.27	0.674 (0.667–0.680)
Bakumondoto	333,204	5,313 (1.59)	391,763	5,921 (1.51)	55	0.158	3.18	0.666 (0.659–0.673)
Shoseiryuto	332,572	4,607 (1.39)	391,796	5,075 (1.30)	66	0.139	2.18	0.687 (0.679–0.694)
Maoto	333,792	3,985 (1.19)	393,610	4,268 (1.08)	57	0.127	1.96	0.622 (0.614–0.631)
Goreisan	335,849	2,745 (0.82)	395,178	3,210 (0.81)	46	0.091	2.24	0.688 (0.678–0.697)
Kikyoto	336,220	2,446 (0.73)	395,700	3,012 (0.76)	43	0.083	2.53	0.688 (0.678–0.698)
Maobushisaishinto	335,812	2,291 (0.68)	395,719	2,563 (0.65)	52	0.080	1.82	0.642 (0.631–0.653)
Shakuyakukanzoto	336,938	1,649 (0.49)	396,715	1,882 (0.47)	56	0.058	1.58	0.726 (0.714–0.738)
Tokishakuyakusan	336,767	1,340 (0.40)	396,574	1,622 (0.41)	57	0.045	1.26	0.855 (0.847–0.862)
Kakkontokasenkyushin’i	336,914	1,610 (0.48)	396,589	1,991 (0.50)	26	0.058	2.40	0.695 (0.683–0.707)

^a^Analyzed in the training set.

^b^Analyzed in the testing set.

**Figure 2.**
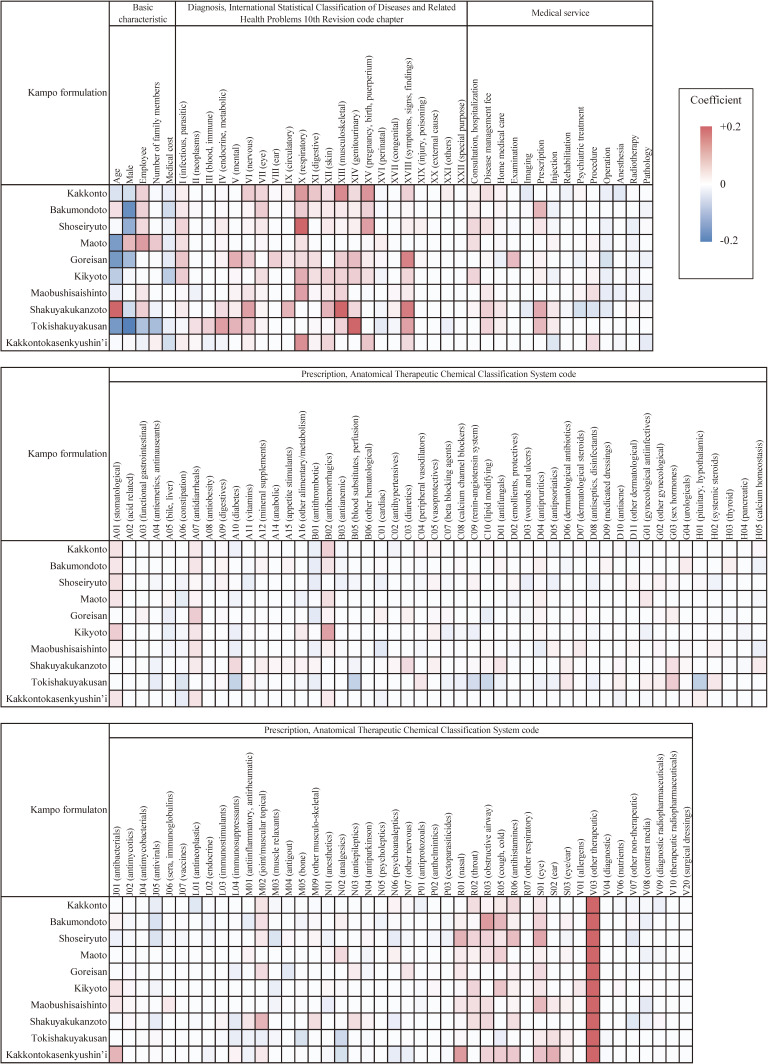
Standardized coefficients of the lasso models for prescription of Kampo formulations

Figure 2 visualizes the standardized coefficients for all 134 variables used in the lasso analyses. Table 4 presents the top 10 variables with the largest absolute values of standardized coefficients. The standardized and non-standardized coefficients are also presented in the table. Different diagnoses, medical services availed, and prescriptions were identified as determinants of the use of various Kampo formulations. However, prescription of drugs classified as ‘all other therapeutic products’ (ATC code, V03) was associated with Kampo use in all models. Male sex was negatively associated with Kampo use in most models. The coefficient for age was negative in most models. Drugs for the respiratory system (ATC codes, R01–R07) was also commonly identified as a significant variable. All non-standardized coefficients from the lasso models and conventional logisitc regression models are presented in eTable 4, eTable 5, eTable 6, eTable 7, and eTable 8. With the exception of two Kampo formulations (maobushisaishinto and kakkontokasenkyushin’i), all variables whose 95% confidence interval did not cross zero were also selected in the lasso models.

**Table 4.** Variables and their coefficients selected in lasso models for Kampo formulation use

Kampo formulation	Variables (standardized coefficient, non-standardized coefficient)									
	1	2	3	4	5	6	7	8	9	10
Kakkonto	*V03* (other therapeutic) (0.364, 0.976)	**XIII** (musculo- skeletal) (0.147, 0.348)	**XV** (pregnancy, birth, puerperium) (0.131, 0.898)	**X** (respiratory) (0.126, 0.252)	**XI** (digestive) (0.065, 0.139)	**VI** (nervous) (0.057, 0.174)	*B02* (antihemorrhagics) (0.054, 0.135)	Employee (0.051, 0.114)	**XVIII** (symptoms, signs, findings) (0.051, 0.122)	Male (−0.047, −0.094)
Bakumondoto	*V03* (other therapeutic) (0.346, 0.928)	Male (−0.178, −0.359)	*R03* (obstructive airway) (0.122, 0.351)	Prescription (0.088, 0.209)	*R05* (cough, cold) (0.085, 0.179)	**VII** (eye) (0.058, 0.124)	**X** (respiratory) (0.058, 0.116)	**XV** (pregnancy, birth, puerperium) (0.051, 0.350)	Medical cost (−0.051, −0.001)	Employee (0.050, 0.113)
Shoseiryuto	*V03* (other therapeutic) (0.467, 1.252)	**X** (respiratory) (0.201, 0.403)	Male (−0.125, −0.252)	**XV** (pregnancy, birth, puerperium) (0.117, 0.802)	*S01* (eye) (0.107, 0.264)	*R01* (nasal) (0.090, 0.254)	*R06* (antihistamines) (0.075, 0.165)	**I** (infectious, parasitic) (0.050, 0.122)	*R03* (obstructive airway) (0.049, 0.141)	*R02* (throat) (0.048, 0.186)
Maoto	*V03* (other therapeutic) (0.268, 0.720)	Age (−0.154, −0.014)	Employee (0.120, 0.269)	Male (0.081, 0.164)	Number of family members (0.061, 0.046)	*R05* (cough, cold) (0.052, 0.108)	*N02* (analgesics) (0.041, 0.089)	*B02* (antihemorrhagics) (0.038, 0.095)	Consultation, hospitalization (0.037, 0.097)	**I** (infectious, parasitic) (0.036, 0.087)
Goreisan	*V03* (other therapeutic) (0.369, 0.988)	Age (−0.160, −0.015)	**XVIII** (symptoms, signs, findings) (0.157, 0.381)	Male (−0.102, −0.207)	**V** (mental) (0.095, 0.347)	Examination (0.078, 0.166)	**XIV** (genito-urinary) (0.076, 0.211)	**I** (infectious, parasitic) (0.064, 0.156)	*A07* (antidiarrheals) (0.058, 0.169)	**VI** (nervous) (0.058, 0.175)
Kikyoto	*V03* (other therapeutic) (0.392, 1.051)	*B02* (antihemorrhagics) (0.115, 0.288)	**X** (respiratory) (0.097, 0.194)	Medical cost (−0.076, −0.001)	Age (−0.076, −0.007)	*R05* (cough, cold) (0.066, 0.137)	**I** (infectious, parasitic) (0.058, 0.142)	*A01* (stomatological) (0.057, 0.231)	**XIV** (genito-urinary) (0.055, 0.152)	**XII** (skin) (0.054, 0.122)
Maobushisaishinto	*V03* (other therapeutic) (0.454, 1.216)	**X** (respiratory) (0.114, 0.229)	*S01* (eye) (0.080, 0.199)	**XIII** (musculo- skeletal) (0.048, 0.113)	*R03* (obstructive airway) (0.037, 0.108)	Procedure (0.037, 0.079)	*R02* (throat) (0.035, 0.137)	*A07* (antidiarrheals) (0.032, 0.092)	**VI** (nervous) (0.029, 0.087)	*V08* (contrast media) (−0.028, −0.161)
Shakuyakukanzoto	Age (0.369, 0.034)	*V03* (other therapeutic) (0.344, 0.921)	**XIII** (musculo- skeletal) (0.212, 0.502)	**XVIII** (symptoms, signs, findings) (0.168, 0.407)	**VI** (nervous) (0.115, 0.347)	Prescription (0.102, 0.240)	**XII** (skin) (0.089, 0.201)	**IX** (circulatory) (0.087, 0.263)	*M02* (joint/muscular topical) (0.087, 0.245)	Disease management fee (0.054, 0.108)
Tokishakuyakusan	Male (−1.290, −2.608)	*V03* (other therapeutic) (0.464, 1.244)	**XIV** (genito-urinary (0.270, 0.746)	Age (−0.160, −0.015)	**IV** (endocrine, metabolic) (0.118, 0.292)	**XVIII** (symptoms, signs, findings) (0.114, 0.276)	Number of family members (−0.099, −0.075)	**V** (mental) (0.089, 0.326)	Employee (−0.089, −0.200)	Prescription (0.081, 0.191)
Kakkonto- kasenkyushin’i,	*V03* (other therapeutic) (0.492, 1.318)	**X** (respiratory) (0.140, 0.280)	*R01* (nasal) (0.123, 0.348)	*S02* (ear) (0.094, 0.361)	*J01* (antibacterials) (0.087, 0.178)	*S01* (eye) (0.067, 0.165)	**XV** (pregnancy, birth, puerperium) (0.065, 0.442)	*R06* (antihistamines) (0.060, 0.134)	*R05* (cough, cold) (0.056, 0.118)	*R03* (obstructive airway) (0.050, 0.143)

Ten variables with large absolute values of standardized coefficients are listed for each model. Bold font indicates the International Statistical Classification of Diseases and Related Health Problems, 10th revision chapter for diagnosis. Italic font indicates the Anatomical Therapeutic Chemical Classification System codes for prescribed drugs.

## DISCUSSION

Using a health insurance claims database, we created machine learning-based models for outpatient prescription of Kampo formulations. Model construction and validation in more than 300,000 individuals showed good performance of the models explaining prescription of 10 Kampo formulations. The characteristics associated with the use of each Kampo formulation were identified.

We used the JMDC Claims Database and analyzed data of enrollees aged 20–59 years. In this population, kakkonto was the most common Kampo formulation prescribed. Bakumondoto, shoseiryuto, and maoto were also used frequently, in approximately 1–2% of enrollees in 1 year. A previous study using the same database have similarly identified these formulations as commonly prescribed and showed that they were used for only a short period in most cases.^11^ Kakkonto and maoto are used for febrile illnesses, such as common cold and influenza, whereas bakumondoto and shoseiryuto are used for bronchitis and asthma. Thus, our analyses were largely reflective of short-term uses for common diseases.

Using the data of the 2018 cohort (training set), we built models that explained outpatient prescription of different Kampo formulations using the data of the preceding year. Prescription of drugs categorized as ‘all other therapeutic products’ (ATC code, V03) was consistently associated with different Kampo formulation uses. Because this category included Kampo products, this result implies that those who received Kampo prescriptions in 1 year were candidates for another Kampo prescription in the following year. Similar to previous studies,^11^^,^^12^^,^^14^ female sex and medical conditions were identified as determinants of Kampo prescriptions. Diseases of the genitourinary system (ICD-10 Chapter XIV, codes N00–N99), which includes gynecological diseases, were associated with the use of tokishakuyakusan. Drugs for the respiratory system (ATC, R01–R07) may reflect repeated respiratory infections and the use of kakkonto, maoto, and kikyoto.

Following model construction, we tested the performance of the models on the 2019 cohort (testing set). Good performance was achieved, indicating that Kampo formulation use can be accurately predicted from background characteristics. The lasso models selected 49 (maoto) to 84 (shakuyakukanzoto) variables from the 134 variables. These models used variables that were statistically significant in the conventional logistic regression models (ie, 95% confidence intervals did not cross zero; the number of variables ranging from 16 for maobushisaishinto to 31 for tokishakuyakusan) plus additional variables and achieved the same or slightly better performance compared with the conventional logistic regression model using all variables. This result showed the advantage of the shrinkage effect by lasso regularization. Nevertheless, the C-statistics was 0.888 at highest (tokishakuyakusan). Background characteristics may have had a small effect on an enrollee having a common acute disease and Kampo formulation being used (eg, kakkonto for common cold). Furthermore, the discriminatory ability decreased when we limited the participants to those who did not receive a prescription of each Kampo formulation during the baseline year. Modeling of new Kampo use among non-users may have been particularly difficult.

In addition to the full lasso models, we created scores using 10 or 5 variables selected by the lasso models. For example, the 5-variable score for tokishakuyakusan was derived as −2.61 × *male* + 1.24 × *V03* + 0.75 × (*N00*–*N99*) − 0.015 × *age* + 0.29 × (*E00*–*E90*), where *V03* represents the prescription of drugs categorized by the ATC codes and (*N00*–*N99*) and (*E00*–*E90*) represent the presence of diagnosis categorized by the respective ICD-10 codes. This variable selection process simplified the model with a small effect on discriminatory ability (eg, 0.883 in the simplified 5-variable score versus 0.888 in the full lasso model for tokishakuyakusan). Similarly, different number of variables can be selected from Table 4 to create a score, depending on the balance between model accuracy and simplicity.

The present study evaluated patient backgrounds based on diagnosis, medical services, and prescription and quantified their effect on future Kampo use. In addition, the scoring system created in this study may be used by the prescribing physicians to identify the possible candidates for each Kampo formulation. However, some caution is necessary when applying the results to clinical setting because the study was conducted using a health insurance claims database. Furthermore, the long-term use of Kampo formulations and their effects were not evaluated. Thus, future studies should investigate the change in symptoms and whether the Kampo formulations were continued, discontinued, or switched to a different Kampo formulation or Western medicine. Traditional diagnosis in Kampo medicine requires pattern classification of patient conditions (*sho*).^15^ However, there has been a concern that general physicians may be prescribing Kampo formulations based on Western diagnoses without consideration of the patterns.^15^ To improve the real-world effectiveness of Kampo formulations, more information regarding the indications for each Kampo drug should be provided to prescribing physicians. Further application of machine learning methods to large-scale, population-representative databases may contribute to the identification of patients who would benefit from pattern-formula matching and may complement the practice of traditional diagnosis.

This study had some limitations. First, it was conducted using a health insurance claims database; thus, data on Kampo formulations purchased as over-the-counter drugs could not be obtained. Second, we analyzed the 10% samples owing to the large computational demand of analyses. Analyses of the entire cohort may result in the creation of more accurate models with more variables. However, considering the good performance of our models and the small differences between the lasso models and the simplified scores, the advantage of using a larger cohort would be trivial. Third, the database consisted of data provided by employer health insurance groups, and data on older individuals were not available. The association between background characteristics and Kampo use may be different in older population. Fourth, there may be a slight underestimation of Kampo users because approximately 8% of individuals were not followed for an entire year after the index date. Fifth, physician- or institution-level data were not considered in the models. This information may have an effect on the prescription of Kampo formulations. Finally, although the models were tested on enrollees in different year, external validation was not conducted. Therefore, further validation in a different population is necessary to confirm the generalizability of models.

In conclusion, lasso models showed good performance in modeling prescription of different Kampo formulations from insurance claims data. The models identified the characteristics that are common to different Kampo formulations and the specific characteristics that were associated with particular Kampo formulations. Further studies using large-scale databases may contribute to the identification of the characteristics of patient who would benefit from using different Kampo formulations.

## SUPPLEMENTARY MATERIAL

eTable 1. Variables of diagnoses, based on International Statistical Classification of Diseases and Related Health Problems, 10th Revision chapters

eTable 2. Variables of medical services, based on Japanese code for reimbursement

eTable 3. Variables of prescriptions, based on Anatomical Therapeutic Chemical Classification System codes

eTable 4. Coefficients of the lasso and conventional logistic regression models for prescription of Kampo formulations (kakkonto, bakumondoto)

eTable 5. Coefficients of the lasso and conventional logistic regression models for prescription of Kampo formulations (shoseiryuito, maoto)

eTable 6. Coefficients of the lasso and conventional logistic regression models for prescription of Kampo formulations (goreisan, kikyoto)

eTable 7. Coefficients of the lasso and conventional logistic regression models for prescription of Kampo formulations (maobushisaishinto, shakuyakukanzoto)

eTable 8. Coefficients of the lasso and conventional logistic regression models for prescription of Kampo formulations (tokishakuyakusan, kakkontokasenkyushin’i)
