# Supplementary material for: Machine Learning-based Models for Outpatient Prescription of Kampo Formulations: An Analysis of a Health Insurance Claims Database
Source: J Epidemiol. 2024 Jan 5;34(1):8–15. doi: 10.2188/jea.JE20220089 (PMC10701253; doi:10.2188/jea.JE20220089)
Supplement: Supplementary file 1 [file je-34-008-s001.pdf]

eTable 1. Variables of diagnoses, based on International Statistical Classification of Diseases and Related Health Problems, 10th Revision chapters

Chapter	Codes	Diagnoses
I	A00–B99	Certain infectious and parasitic diseases
II	C00–D48	Neoplasms
III	D50–D89	Diseases of the blood and blood-forming organs and certain disorders involving the immune mechanism
IV	E00–E90	Endocrine, nutritional and metabolic diseases
V	F00–F99	Mental and behavioral disorders
VI	G00–G99	Diseases of the nervous system
VII	H00–H59	Diseases of the eye and adnexa
VIII	H60–H95	Diseases of the ear and mastoid process
IX	I00–I99	Diseases of the circulatory system
X	J00–J99	Diseases of the respiratory system
XI	K00–K93	Diseases of the digestive system
XII	L00–L99	Diseases of the skin and subcutaneous tissue
XIII	M00–M99	Diseases of the musculoskeletal system and connective tissue
XIV	N00–N99	Diseases of the genitourinary system
XV	O00–O99	Pregnancy, childbirth and the puerperium
XVI	P00–P96	Certain conditions originating in the perinatal period
XVII	Q00–Q99	Congenital malformations, deformations and chromosomal abnormalities
XVIII	R00–R99	Symptoms, signs and abnormal clinical and laboratory findings, not elsewhere classified
XIX	S00–T98	Injury, poisoning and certain other consequences of external causes
XX	V01–Y98	External causes of morbidity and mortality
XXI	Z00–Z99	Factors influencing health status and contact with health services
XXII	U00–U99	Codes for special purposes

eTable 2. Variables of medical services, based on Japanese code for reimbursement

Codes	Medical services
A	Consultation, hospitalization
B	Disease management fee
C	Home medical care
D	Examination
E	Imaging
F	Prescription
G	Injection
H	Rehabilitation
I	Psychiatric treatment
J	Procedure
K	Operation
L	Anesthesia
M	Radiotherapy
N	Pathology

eTable 3. Variables of prescriptions, based on Anatomical Therapeutic Chemical Classification System codes

Codes	Drugs	Codes	Drugs
A01	Stomatological preparations	D01	Antifungals for dermatological use
A02	Drugs for acid related disorders	D02	Emollients and protectives
A03	Drugs for functional gastrointestinal disorders	D03	Preparations for treatment of wounds and ulcers
A04	Antiemetics and antinauseants	D04	Antipruritics, including antihistamines, anesthetics, etc.
A05	Bile and liver therapy	D05	Antipsoriatics
A06	Drugs for constipation	D06	Antibiotics and chemotherapeutics for dermatological use
A07	Antidiarrheals, intestinal antiinflammatory/antiinfective agents	D07	Corticosteroids, dermatological preparations
A08	Antiobesity preparations, excl. diet products	D08	Antiseptics and disinfectants
A09	Digestives, including enzymes	D09	Medicated dressings
A10	Drugs used in diabetes	D10	Anti-acne preparations
A11	Vitamins	D11	Other dermatological preparations
A12	Mineral supplements	G01	Gynecological antiinfectives and antiseptics
A14	Anabolic agents for systemic use	G02	Other gynecologicals
A15	Appetite stimulants	G03	Sex hormones and modulators of the genital system
A16	Other alimentary tract and metabolism products	G04	Urologicals
B01	Antithrombotic agents	H01	Pituitary and hypothalamic hormones and analogues
B02	Antihemorrhagics	H02	Corticosteroids for systemic use
B03	Antianemic preparations	H03	Thyroid therapy
B05	Blood substitutes and perfusion solutions	H04	Pancreatic hormones
B06	Other hematological agents	H05	Calcium homeostasis
C01	Cardiac therapy	J01	Antibacterials for systemic use
C02	Antihypertensives	J02	Antimycotics for systemic use
C03	Diuretics	J04	Antimycobacterials
C04	Peripheral vasodilators	J05	Antivirals for systemic use
C05	Vasoprotectives	J06	Immune sera and immunoglobulins
C07	Beta blocking agents	J07	Vaccines
C08	Calcium channel blockers	L01	Antineoplastic agents
C09	Agents acting on the renin-angiotensin system	L02	Endocrine therapy
C10	Lipid modifying agents	L03	Immunostimulants
		L04	Immunosuppressants

eTable 3 (continued). Variables of prescriptions, based on Anatomical Therapeutic Chemical Classification System codes

Codes	Drugs	Codes	Drugs
M01	Antiinflammatory and antirheumatic products	R02	Throat preparations
M02	Topical products for joint and muscular pain	R03	Drugs for obstructive airway diseases
M03	Muscle relaxants	R05	Cough and cold preparations
M04	Antigout preparations	R06	Antihistamines for systemic use
M05	Drugs for treatment of bone diseases	R07	Other respiratory system products
M09	Other drugs for disorders of the musculo-skeletal system	S01	Ophthalmologicals
N01	Anesthetics	S02	Otologicals
N02	Analgesics	S03	Ophthalmological and otological preparations
N03	Antiepileptics	V01	Allergens
N04	Anti-parkinson drugs	V03	All other therapeutic products
N05	Psycholeptics	V04	Diagnostic agents
N06	Psychoanaleptics	V06	General nutrients
N07	Other nervous system drugs	V07	All other non-therapeutic products
P01	Antiprotozoals	V08	Contrast media
P02	Athelmintics	V09	Diagnostic radiopharmaceuticals
P03	Ectoparasiticides, including scabicides, insecticides and repellents	V10	Therapeutic radiopharmaceuticals
R01	Nasal preparations	V20	Surgical dressings

eTable 4. Coefficients of the lasso and conventional logistic regression models for prescription of Kampo formulations (kakkonto, bakumondoto)

Variable	Kakkonto		Bakumondoto	
	Lasso	Conventional logistic [95% confidence interval]	Lasso	Conventional logistic [95% confidence interval]
Basic characteristics				
Age	-0.004	-0.005 [-0.008 to -0.003]	0.003	0.004 [0.001–0.007]
Male	-0.094	-0.134 [-0.197 to -0.070]	-0.359	-0.407 [-0.478 to -0.336]
Employee	0.114	0.169 [0.097–0.242]	0.113	0.171 [0.090–0.252]
Number of family members	-0.002	0.002 [-0.020 to 0.024]	–	0.004 [-0.021 to 0.028]
Medical cost	0.000	0.000 [-0.001 to 0.000]	-0.001	-0.001 [-0.002 to -0.001]
Diagnosis, International Statistical Classification of Diseases and Related Health Problems 10th Revision chapter				
I (infectious, parasitic)	0.041	0.059 [-0.004 to 0.123]	0.062	0.069 [-0.003 to 0.141]
II (neoplasms)	–	0.046 [-0.030 to 0.122]	0.016	0.062 [-0.023 to 0.148]
III (blood, immune)	–	-0.014 [-0.104 to 0.077]	–	-0.009 [-0.115 to 0.096]
IV (endocrine, metabolic)	0.008	0.017 [-0.049 to 0.083]	0.008	0.019 [-0.057 to 0.095]
V (mental)	–	-0.038 [-0.142 to 0.067]	–	0.006 [-0.115 to 0.128]
VI (nervous)	0.174	0.180 [0.108–0.252]	0.028	0.072 [-0.014 to 0.157]
VII (eye)	0.096	0.107 [0.039–0.174]	0.124	0.137 [0.060–0.213]
VIII (ear)	0.011	0.042 [-0.039 to 0.123]	–	-0.057 [-0.150 to 0.035]
IX (circulatory)	0.042	0.073 [-0.013 to 0.158]	0.011	0.027 [-0.072 to 0.127]
X (respiratory)	0.252	0.250 [0.172–0.328]	0.116	0.115 [0.025–0.205]
XI (digestive)	0.139	0.161 [0.100–0.222]	0.045	0.075 [0.006–0.143]
XII (skin)	0.075	0.071 [0.000–0.142]	0.052	
XIII (musculoskeletal)	0.348	0.376 [0.312–0.440]	0.052	0.066 [-0.009 to 0.141]
XIV (genitourinary)	0.111	0.119 [0.052–0.186]	0.051	0.070 [-0.007 to 0.146]
XV (pregnancy, birth, puerperium)	0.898	0.916 [0.788–1.044]	0.350	0.410 [0.242–0.577]
XVI (perinatal)	-0.119	-0.229 [-0.567 to 0.109]	-0.108	-0.289 [-0.768 to 0.189]
XVII (congenital)	–	0.063 [-0.110 to 0.235]	0.011	0.067 [-0.129 to 0.264]
XVIII (symptoms, signs, findings)	0.122	0.125 [0.071–0.180]	0.016	0.024 [-0.039 to 0.086]
XIX (injury, poisoning)	–	-0.017 [-0.086 to 0.052]	–	-0.009 [-0.087 to 0.070]
XX (external cause)	–	–	–	–
XXI (others)	–	–	–	–
XXII (special purpose)	0.166	0.239 [0.085–0.393]	–	-0.015 [-0.217 to 0.186]
Medical service				
Consultation, hospitalization	0.041	0.123 [-0.043 to 0.290]	0.049	0.100 [-0.089 to 0.289]
Disease management fee	0.069	0.081 [0.025–0.138]	–	0.003 [-0.058 to 0.065]
Home medical care	0.110	0.198 [0.035–0.361]	0.072	0.137 [-0.066 to 0.340]
Examination	–	-0.053 [-0.128 to 0.022]	–	-0.032 [-0.115 to 0.051]
Imaging	-0.058	-0.096 [-0.155 to -0.037]	0.010	0.032 [-0.034 to 0.098]
Prescription	–	-0.007 [-0.152 to 0.138]	0.209	0.213 [0.049–0.378]
Injection	–	0.021 [-0.056 to 0.098]	-0.040	-0.064 [-0.154 to 0.026]
Rehabilitation	-0.089	-0.125 [-0.249 to 0.000]	–	0.040 [-0.100 to 0.179]
Psychiatric treatment	0.028	0.053 [-0.083 to 0.189]	–	0.008 [-0.156 to 0.173]
Procedure	–	-0.014 [-0.075 to 0.046]	0.002	0.028 [-0.040 to 0.097]
Operation	-0.074	-0.082 [-0.187 to 0.024]	–	-0.030 [-0.152 to 0.091]
Anesthesia	-0.157	-0.147 [-0.280 to -0.014]	–	0.039 [-0.115 to 0.193]
Radiotherapy	-0.008	-0.111 [-0.865 to 0.643]	–	0.224 [-0.480 to 0.927]
Pathology	–	-0.029 [-0.109 to 0.052]	-0.035	-0.077 [-0.170 to 0.016]

– : omitted variable

eTable 4 (continued). Coefficients of the lasso and conventional logistic regression models for prescription of Kampo formulations (kakkonto, bakumondoto)

Variable	Kakkonto		Bakumondoto	
	Lasso	Conventional logistic [95% confidence interval]	Lasso	Conventional logistic [95% confidence interval]
Prescription, Anatomical Therapeutic Chemical Classification System code				
A01 (stomatological)	0.127	0.141 [0.067–0.214]	0.109	0.121 [0.037–0.204]
A02 (acid related)	–	-0.021 [-0.083 to 0.040]	–	-0.038 [-0.108 to 0.031]
A03 (functional gastrointestinal)	–	0.007 [-0.060 to 0.075]	0.002	0.038 [-0.040 to 0.116]
A04 (antiemetics, antinauseants)	-0.013	-0.079 [-0.977 to 0.819]	–	-0.224 [-1.157 to 0.710]
A05 (bile, liver)	-0.022	-0.096 [-0.270 to 0.078]	–	-0.033 [-0.236 to 0.171]
A06 (constipation)	–	-0.018 [-0.113 to 0.076]	-0.008	-0.041 [-0.154 to 0.072]
A07 (antidiarrheals)	0.068	0.060 [-0.008 to 0.128]	0.081	0.087 [0.009–0.164]
A08 (antiobesity)	-0.513	–	–	0.372 [-1.664 to 2.407]
A09 (digestives)	0.046	0.085 [-0.064 to 0.235]	0.095	0.145 [-0.024 to 0.314]
A10 (diabetes)	-0.099	-0.210 [-0.369 to -0.052]	–	0.028 [-0.147 to 0.203]
A11 (vitamins)	–	0.000 [-0.085 to 0.086]	-0.019	-0.058 [-0.160 to 0.045]
A12 (mineral supplements)	-0.036	-0.139 [-0.471 to 0.194]	0.180	0.361 [0.027–0.696]
A14 (anabolic)	–	–	–	–
A15 (appetite stimulants)	–	-0.511 [-2.511 to 1.489]	0.975	1.150 [0.080–2.220]
A16 (other alimentary/metabolism)	–	0.058 [-0.220 to 0.336]	–	-0.051 [-0.393 to 0.290]
B01 (antithrombotic)	-0.119	-0.147 [-0.283 to -0.011]	-0.150	-0.220 [-0.390 to -0.050]
B02 (antihemorrhagics)	0.135	0.147 [0.091–0.203]	0.073	0.084 [0.021–0.147]
B03 (antianemic)	-0.029	-0.063 [-0.156 to 0.029]	-0.024	-0.073 [-0.184 to 0.037]
B05 (blood substitutes, perfusion)	–	0.020 [-0.051 to 0.090]	–	-0.014 [-0.094 to 0.066]
B06 (other hematological)	–	–	–	–
C01 (cardiac)	-0.016	-0.049 [-0.161 to 0.063]	0.017	0.135 [0.008–0.261]
C02 (antihypertensives)	-0.170	-0.345 [-0.727 to 0.037]	–	-0.016 [-0.426 to 0.393]
C03 (diuretics)	–	0.081 [-0.139 to 0.301]	0.019	0.127 [-0.129 to 0.383]
C04 (peripheral vasodilators)	0.078	0.128 [-0.048 to 0.303]	-0.032	-0.124 [-0.357 to 0.110]
C05 (vasoprotectives)	0.021	0.036 [-0.039 to 0.111]	–	0.005 [-0.081 to 0.091]
C07 (beta blocking agents)	–	-0.081 [-0.261 to 0.099]	–	0.021 [-0.189 to 0.230]
C08 (calcium channel blockers)	–	-0.007 [-0.125 to 0.111]	0.018	0.053 [-0.084 to 0.191]
C09 (renin-angiotensin system)	0.007	0.050 [-0.067 to 0.167]	–	-0.053 [-0.191 to 0.086]
C10 (lipid modifying)	0.032	0.082 [-0.020 to 0.183]	–	0.014 [-0.106 to 0.133]
D01 (antifungals)	–	-0.033 [-0.132 to 0.065]	0.043	0.079 [-0.030 to 0.189]
D02 (emollients, protectives)	–	-0.008 [-0.102 to 0.087]	–	-0.016 [-0.123 to 0.091]
D03 (wounds and ulcers)	-0.006	-0.092 [-0.285 to 0.101]	0.027	0.096 [-0.109 to 0.301]
D04 (antipruritics)	0.012	0.062 [-0.092 to 0.216]	0.145	0.208 [0.037–0.379]
D05 (antipsoriatics)	-0.092	-0.245 [-0.574 to 0.083]	–	-0.038 [-0.371 to 0.295]
D06 (dermatological antibiotics)	0.006	0.043 [-0.048 to 0.133]	-0.037	-0.087 [-0.195 to 0.021]
D07 (dermatological steroids)	0.007	0.027 [-0.049 to 0.102]	–	0.001 [-0.085 to 0.087]
D08 (antiseptics, disinfectants)	-0.029	-0.059 [-0.201 to 0.082]	–	-0.017 [-0.180 to 0.145]
D09 (medicated dressings)	–	0.509 [-1.601 to 2.619]	1.713	2.097 [0.511–3.684]
D10 (antiacne)	0.023	0.037 [-0.070 to 0.144]	–	-0.013 [-0.137 to 0.111]
D11 (other dermatological)	-0.067	-0.144 [-0.278 to -0.010]	0.004	0.050 [-0.093 to 0.192]
G01 (gynecological antiinfectives)	–	-0.044 [-0.175 to 0.088]	-0.006	-0.104 [-0.260 to 0.052]
G02 (other gynecological)	–	0.041 [-0.131 to 0.212]	–	0.055 [-0.173 to 0.283]
G03 (sex hormones)	0.011	0.017 [-0.096 to 0.130]	–	-0.020 [-0.153 to 0.113]
G04 (urologicals)	0.027	0.097 [-0.082 to 0.276]	0.191	0.247 [0.045–0.449]
H01 (pituitary, hypothalamic)	–	-0.055 [-0.264 to 0.154]	–	-0.038 [-0.315 to 0.238]
H02 (systemic steroids)	–	-0.015 [-0.081 to 0.052]	–	-0.048 [-0.122 to 0.027]
H03 (thyroid)	–	-0.030 [-0.223 to 0.162]	0.146	0.178 [-0.022 to 0.379]
H04 (pancreatic)	–	-0.045 [-0.429 to 0.339]	–	0.064 [-0.373 to 0.501]
H05 (calcium homeostasis)	0.316	0.609 [-0.011 to 1.228]	-0.627	-1.526 [-3.527 to 0.476]

– : omitted variable

eTable 4 (continued). Coefficients of the lasso and conventional logistic regression models for prescription of Kampo formulations (kakkonto, bakumondoto)

Variable	Kakkonto		Bakumondoto	
	Lasso	Conventional logistic [95% confidence interval]	Lasso	Conventional logistic [95% confidence interval]
Prescription, Anatomical Therapeutic Chemical Classification System code				
J01 (antibacterials)	0.003	0.021 [-0.040 to 0.082]	0.023	0.047 [-0.022 to 0.115]
J02 (antimycotics)	–	-0.159 [-0.745 to 0.428]	-0.162	-0.494 [-1.252 to 0.264]
J04 (antimycobacterials)	–	0.150 [-0.864 to 1.164]	–	-0.257 [-1.664 to 1.150]
J05 (antivirals)	-0.069	-0.105 [-0.175 to -0.035]	-0.133	-0.172 [-0.252 to -0.092]
J06 (sera, immunoglobulins)	–	0.159 [-0.254 to 0.572]	-0.001	-0.179 [-0.737 to 0.379]
J07 (vaccines)	–	-0.050 [-0.693 to 0.592]	-0.008	-0.356 [-1.249 to 0.537]
L01 (antineoplastic)	-0.494	-0.575 [-1.074 to -0.075]	–	-0.011 [-0.468 to 0.446]
L02 (endocrine)	-0.043	-0.146 [-0.465 to 0.174]	-0.121	-0.290 [-0.656 to 0.077]
L03 (immunostimulants)	–	-0.017 [-0.958 to 0.923]	–	0.419 [-0.519 to 1.357]
L04 (immunosuppressants)	–	-0.011 [-0.284 to 0.262]	-0.001	-0.063 [-0.424 to 0.297]
M01 (antiinflammatory, antirheumatic)	–	-0.041 [-0.098 to 0.017]	-0.052	-0.098 [-0.162 to -0.034]
M02 (joint/muscular topical)	0.084	0.132 [0.061 to 0.203]	0.094	0.114 [0.030 to 0.198]
M03 (muscle relaxants)	-0.033	-0.059 [-0.159 to 0.041]	-0.008	-0.056 [-0.176 to 0.064]
M04 (antigout)	-0.015	-0.100 [-0.239 to 0.038]	–	0.017 [-0.148 to 0.181]
M05 (bone)	–	-0.032 [-0.395 to 0.331]	-0.184	-0.354 [-0.868 to 0.160]
M09 (other musculo-skeletal)	-0.038	-0.132 [-0.361 to 0.097]	0.056	0.141 [-0.103 to 0.385]
N01 (anesthetics)	-0.008	-0.011 [-0.097 to 0.076]	–	0.019 [-0.080 to 0.118]
N02 (analgesics)	–	0.010 [-0.045 to 0.066]	0.034	0.053 [-0.010 to 0.115]
N03 (antiepileptics)	–	0.004 [-0.117 to 0.125]	–	0.000 [-0.150 to 0.150]
N04 (antiparkinson)	–	0.043 [-0.217 to 0.304]	-0.006	-0.141 [-0.502 to 0.221]
N05 (psycholeptics)	–	0.028 [-0.058 to 0.114]	-0.037	-0.073 [-0.175 to 0.030]
N06 (psychoanaleptics)	0.075	0.088 [-0.038 to 0.214]	-0.010	-0.069 [-0.227 to 0.089]
N07 (other nervous)	0.040	0.074 [-0.061 to 0.210]	–	-0.063 [-0.229 to 0.103]
P01 (antiprotozoals)	-0.736	-1.661 [-3.648 to 0.326]	–	0.189 [-0.985 to 1.363]
P02 (anthelmintics)	0.073	0.474 [-0.751 to 1.700]	–	-0.441 [-2.447 to 1.565]
P03 (ectoparasiticides)	–	-0.031 [-0.158 to 0.097]	-0.031	-0.096 [-0.247 to 0.055]
R01 (nasal)	0.006	0.028 [-0.036 to 0.091]	0.044	0.054 [-0.016 to 0.125]
R02 (throat)	0.149	0.156 [0.084 to 0.227]	0.017	0.033 [-0.050 to 0.116]
R03 (obstructive airway)	0.035	0.044 [-0.016 to 0.104]	0.351	0.353 [0.288 to 0.417]
R05 (cough, cold)	0.051	0.058 [-0.006 to 0.123]	0.179	0.177 [0.102 to 0.252]
R06 (antihistamines)	0.090	0.093 [0.038 to 0.148]	0.034	0.036 [-0.026 to 0.099]
R07 (other respiratory)	–	0.344 [-0.837 to 1.526]	–	-0.484 [-2.474 to 1.506]
S01 (eye)	0.062	0.068 [-0.004 to 0.141]	0.119	0.120 [0.039 to 0.200]
S02 (ear)	–	-0.006 [-0.092 to 0.080]	–	0.016 [-0.077 to 0.108]
S03 (eye/ear)	–	-0.035 [-0.116 to 0.046]	0.044	0.036 [-0.052 to 0.123]
V01 (allergens)	–	0.009 [-0.497 to 0.515]	–	0.083 [-0.456 to 0.621]
V03 (other therapeutic)	0.976	0.978 [0.926 to 1.031]	0.928	0.933 [0.874 to 0.992]
V04 (diagnostic)	–	-0.026 [-0.150 to 0.099]	–	0.018 [-0.130 to 0.165]
V06 (nutrients)	-0.038	-0.077 [-0.216 to 0.062]	–	-0.001 [-0.172 to 0.170]
V07 (other non-therapeutic)	-0.029	-0.053 [-0.134 to 0.029]	0.028	0.047 [-0.041 to 0.135]
V08 (contrast media)	-0.028	-0.042 [-0.166 to 0.083]	-0.090	-0.148 [-0.297 to 0.002]
V09 (diagnostic radiopharmaceuticals)	-0.310	-0.457 [-0.971 to 0.058]	–	0.129 [-0.339 to 0.597]
V10 (therapeutic radiopharmaceuticals)	–	–	–	–
V20 (surgical dressings)	–	–	–	–

– : omitted variable

eTable 5. Coefficients of the lasso and conventional logistic regression models for prescription of Kampo formulations (shoseiryuito, maoto)

Variable	Shoseiryuito		Maoto	
	Lasso	Conventional logistic [95% confidence interval]	Lasso	Conventional logistic [95% confidence interval]
Basic characteristics				
Age	0.000	-0.002 [-0.005 to 0.001]	-0.014	-0.017 [-0.020 to -0.013]
Male	-0.252	-0.305 [-0.377 to -0.232]	0.164	0.186 [0.102–0.271]
Employee	0.092	0.146 [0.063–0.229]	0.269	0.350 [0.250–0.450]
Number of family members	–	0.006 [-0.020 to 0.031]	0.046	0.073 [0.047–0.100]
Medical cost	0.000	0.000 [-0.001 to 0.000]	0.000	-0.001 [-0.002 to 0.000]
Diagnosis, International Statistical Classification of Diseases and Related Health Problems 10th Revision chapter				
I (infectious, parasitic)	0.122	0.135 [0.063–0.207]	0.087	0.119 [0.029–0.208]
II (neoplasms)	–	-0.007 [-0.094 to 0.079]	–	-0.029 [-0.149 to 0.090]
III (blood, immune)	-0.017	-0.052 [-0.158 to 0.053]	–	0.028 [-0.115 to 0.172]
IV (endocrine, metabolic)	0.051	0.080 [0.005–0.155]	0.019	0.076 [-0.019 to 0.172]
V (mental)	–	0.003 [-0.117 to 0.123]	–	-0.095 [-0.263 to 0.074]
VI (nervous)	0.026	0.062 [-0.023 to 0.148]	–	-0.006 [-0.117 to 0.105]
VII (eye)	0.051	0.068 [-0.010 to 0.147]	0.016	0.036 [-0.055 to 0.128]
VIII (ear)	–	0.003 [-0.089 to 0.095]	–	0.030 [-0.087 to 0.147]
IX (circulatory)	–	0.017 [-0.084 to 0.118]	–	-0.068 [-0.201 to 0.065]
X (respiratory)	0.403	0.420 [0.328–0.512]	0.062	0.053 [-0.052 to 0.158]
XI (digestive)	0.003	0.037 [-0.033 to 0.106]	–	0.010 [-0.073 to 0.094]
XII (skin)	0.050	0.069 [-0.012 to 0.149]	–	-0.016 [-0.119 to 0.087]
XIII (musculoskeletal)	0.025	0.043 [-0.031 to 0.118]	–	-0.012 [-0.106 to 0.083]
XIV (genitourinary)	0.046	0.074 [-0.002 to 0.151]	–	0.004 [-0.098 to 0.106]
XV (pregnancy, birth, puerperium)	0.802	0.801 [0.657–0.946]	0.029	0.169 [-0.072 to 0.410]
XVI (perinatal)	–	-0.061 [-0.412 to 0.289]	0.409	0.559 [0.075–1.043]
XVII (congenital)	–	-0.053 [-0.258 to 0.152]	–	-0.007 [-0.285 to 0.271]
XVIII (symptoms, signs, findings)	0.040	0.057 [-0.005 to 0.120]	0.002	0.025 [-0.052 to 0.103]
XIX (injury, poisoning)	0.047	0.083 [0.005–0.160]	–	0.012 [-0.087 to 0.112]
XX (external cause)	–	–	–	–
XXI (others)	–	–	–	–
XXII (special purpose)	–	0.042 [-0.148 to 0.231]	–	0.024 [-0.244 to 0.293]
Medical service				
Consultation, hospitalization	0.116	0.220 [0.012–0.427]	0.097	0.150 [-0.033 to 0.333]
Disease management fee	0.068	0.084 [0.021–0.147]	–	-0.049 [-0.122 to 0.024]
Home medical care	0.040	0.130 [-0.069 to 0.329]	0.039	0.249 [0.000–0.498]
Examination	-0.010	-0.097 [-0.181 to -0.013]	–	-0.006 [-0.100 to 0.088]
Imaging	–	-0.029 [-0.096 to 0.038]	–	-0.035 [-0.118 to 0.047]
Prescription	0.062	0.070 [-0.111 to 0.250]	–	0.004 [-0.160 to 0.168]
Injection	-0.020	-0.049 [-0.138 to 0.040]	0.032	0.062 [-0.049 to 0.174]
Rehabilitation	–	-0.004 [-0.149 to 0.141]	–	-0.006 [-0.190 to 0.179]
Psychiatric treatment	–	0.048 [-0.114 to 0.210]	0.008	0.131 [-0.084 to 0.346]
Procedure	–	-0.011 [-0.080 to 0.058]	0.020	0.046 [-0.038 to 0.130]
Operation	-0.051	-0.063 [-0.184 to 0.059]	-0.054	-0.132 [-0.290 to 0.027]
Anesthesia	–	0.042 [-0.114 to 0.198]	–	-0.100 [-0.301 to 0.101]
Radiotherapy	-0.580	-1.003 [-2.181 to 0.175]	-0.300	-1.357 [-3.381 to 0.667]
Pathology	-0.008	-0.030 [-0.122 to 0.063]	–	0.023 [-0.101 to 0.147]

– : omitted variable

eTable 5 (continued). Coefficients of the lasso and conventional logistic regression models for prescription of Kampo formulations (shoseiryuito, maoto)

Variable	Shoseiryuito		Maoto	
	Lasso	Conventional logistic [95% confidence interval]	Lasso	Conventional logistic [95% confidence interval]
Prescription, Anatomical Therapeutic Chemical Classification System code				
A01 (stomatological)	0.140	0.158 [0.076–0.240]	0.121	0.142 [0.036–0.247]
A02 (acid related)	–	-0.033 [-0.103 to 0.037]	–	-0.061 [-0.146 to 0.024]
A03 (functional gastrointestinal)	–	-0.003 [-0.081 to 0.074]	–	-0.030 [-0.129 to 0.068]
A04 (antiemetics, antinauseants)	-0.173	-0.386 [-1.353 to 0.581]	–	0.224 [-1.069 to 1.516]
A05 (bile, liver)	–	-0.030 [-0.227 to 0.167]	0.051	0.166 [-0.069 to 0.401]
A06 (constipation)	-0.049	-0.070 [-0.182 to 0.042]	-0.121	-0.222 [-0.377 to -0.068]
A07 (antidiarrheals)	0.019	0.035 [-0.042 to 0.113]	0.037	0.043 [-0.055 to 0.140]
A08 (antiobesity)	-0.504	–	0.094	1.050 [-0.982 to 3.082]
A09 (digestives)	0.098	0.140 [-0.029 to 0.308]	–	-0.007 [-0.236 to 0.223]
A10 (diabetes)	-0.006	-0.114 [-0.298 to 0.070]	-0.077	-0.311 [-0.552 to -0.071]
A11 (vitamins)	-0.093	-0.137 [-0.239 to -0.035]	–	0.062 [-0.069 to 0.193]
A12 (mineral supplements)	–	0.027 [-0.349 to 0.403]	–	-0.172 [-0.735 to 0.392]
A14 (anabolic)	–	–	–	–
A15 (appetite stimulants)	0.005	0.453 [-1.000 to 1.906]	–	0.325 [-1.672 to 2.323]
A16 (other alimentary/metabolism)	–	0.032 [-0.299 to 0.362]	-0.224	-0.607 [-1.208 to -0.007]
B01 (antithrombotic)	-0.155	-0.197 [-0.363 to -0.032]	-0.020	-0.097 [-0.312 to 0.118]
B02 (antihemorrhagics)	–	0.013 [-0.050 to 0.075]	0.095	0.105 [0.027–0.182]
B03 (antianemic)	-0.054	-0.076 [-0.186 to 0.035]	–	0.004 [-0.142 to 0.150]
B05 (blood substitutes, perfusion)	-0.022	-0.021 [-0.101 to 0.059]	–	0.009 [-0.091 to 0.109]
B06 (other hematological)	–	–	–	–
C01 (cardiac)	-0.065	-0.067 [-0.199 to 0.066]	–	-0.034 [-0.205 to 0.137]
C02 (antihypertensives)	–	0.002 [-0.389 to 0.394]	–	-0.042 [-0.602 to 0.518]
C03 (diuretics)	–	0.045 [-0.218 to 0.309]	–	0.102 [-0.246 to 0.449]
C04 (peripheral vasodilators)	-0.054	-0.130 [-0.359 to 0.099]	–	0.127 [-0.160 to 0.413]
C05 (vasoprotectives)	0.034	0.048 [-0.036 to 0.132]	–	0.037 [-0.075 to 0.148]
C07 (beta blocking agents)	-0.042	-0.158 [-0.382 to 0.066]	–	0.026 [-0.245 to 0.298]
C08 (calcium channel blockers)	–	0.030 [-0.109 to 0.169]	–	0.032 [-0.151 to 0.214]
C09 (renin-angiotensin system)	0.054	0.086 [-0.053 to 0.224]	–	0.058 [-0.123 to 0.239]
C10 (lipid modifying)	–	-0.017 [-0.137 to 0.104]	0.001	0.110 [-0.040 to 0.261]
D01 (antifungals)	–	0.024 [-0.085 to 0.133]	–	-0.099 [-0.243 to 0.046]
D02 (emollients, protectives)	0.040	0.072 [-0.032 to 0.175]	-0.034	-0.125 [-0.270 to 0.019]
D03 (wounds and ulcers)	-0.196	-0.303 [-0.538 to -0.069]	–	0.105 [-0.168 to 0.378]
D04 (antipruritics)	0.034	0.087 [-0.083 to 0.258]	0.125	0.250 [0.026–0.473]
D05 (antipsoriatics)	-0.223	-0.393 [-0.787 to 0.002]	–	0.018 [-0.400 to 0.436]
D06 (dermatological antibiotics)	–	0.028 [-0.075 to 0.131]	-0.004	-0.095 [-0.235 to 0.044]
D07 (dermatological steroids)	–	-0.032 [-0.117 to 0.054]	–	0.029 [-0.082 to 0.139]
D08 (antiseptics, disinfectants)	-0.094	-0.140 [-0.308 to 0.029]	–	0.023 [-0.183 to 0.230]
D09 (medicated dressings)	-0.267	–	–	–
D10 (antiacne)	–	-0.014 [-0.136 to 0.108]	–	-0.004 [-0.161 to 0.152]
D11 (other dermatological)	0.062	0.091 [-0.046 to 0.228]	–	-0.038 [-0.228 to 0.153]
G01 (gynecological antiinfectives)	-0.017	-0.093 [-0.240 to 0.053]	0.190	0.282 [0.082–0.482]
G02 (other gynecological)	0.122	0.161 [-0.029 to 0.351]	–	-0.100 [-0.430 to 0.231]
G03 (sex hormones)	0.033	0.046 [-0.080 to 0.171]	–	-0.057 [-0.249 to 0.136]
G04 (urologicals)	–	-0.023 [-0.248 to 0.202]	–	-0.032 [-0.322 to 0.258]
H01 (pituitary, hypothalamic)	0.064	0.140 [-0.089 to 0.369]	–	0.014 [-0.370 to 0.398]
H02 (systemic steroids)	-0.034	-0.060 [-0.134 to 0.015]	–	-0.013 [-0.107 to 0.081]
H03 (thyroid)	–	-0.038 [-0.256 to 0.179]	–	-0.078 [-0.399 to 0.244]
H04 (pancreatic)	–	0.073 [-0.375 to 0.521]	–	-0.373 [-1.081 to 0.334]
H05 (calcium homeostasis)	–	0.062 [-0.828 to 0.953]	-0.313	–

– : omitted variable

eTable 5 (continued). Coefficients of the lasso and conventional logistic regression models for prescription of Kampo formulations (shoseiryuito, maoto)

Variable	Shoseiryuito		Maoto	
	Lasso	Conventional logistic [95% confidence interval]	Lasso	Conventional logistic [95% confidence interval]
Prescription, Anatomical Therapeutic Chemical Classification System code				
J01 (antibacterials)	-0.032	-0.087 [-0.155 to -0.018]	–	-0.003 [-0.086,0.080]
J02 (antimycotics)	-0.143	-0.367 [-1.079 to 0.345]	–	0.385 [-0.257,1.026]
J04 (antimycobacterials)	–	0.194 [-0.972 to 1.359]	–	0.225 [-1.183,1.633]
J05 (antivirals)	-0.128	-0.154 [-0.232 to -0.075]	–	-0.057 [-0.151,0.038]
J06 (sera, immunoglobulins)	-0.136	-0.285 [-0.826 to 0.256]	–	0.012 [-0.620,0.645]
J07 (vaccines)	-0.187	-0.617 [-1.614 to 0.380]	–	-0.276 [-1.272,0.719]
L01 (antineoplastic)	–	0.136 [-0.287 to 0.559]	–	-0.173 [-0.809,0.462]
L02 (endocrine)	-0.080	-0.183 [-0.552 to 0.186]	–	0.191 [-0.308,0.689]
L03 (immunostimulants)	–	-0.119 [-1.276 to 1.037]	–	-0.236 [-1.816,1.344]
L04 (immunosuppressants)	–	-0.054 [-0.400 to 0.292]	–	-0.127 [-0.595,0.340]
M01 (antiinflammatory, antirheumatic)	–	0.012 [-0.053 to 0.076]	0.029	0.064 [-0.015,0.143]
M02 (joint/muscular topical)	0.068	0.088 [0.004–0.173]	0.000	0.065 [-0.043,0.174]
M03 (muscle relaxants)	-0.185	-0.220 [-0.347 to -0.093]	–	0.020 [-0.132,0.172]
M04 (antigout)	0.049	0.102 [-0.059 to 0.262]	–	-0.057 [-0.248,0.135]
M05 (bone)	-0.020	-0.149 [-0.626 to 0.329]	–	-0.081 [-0.764,0.603]
M09 (other musculo-skeletal)	0.112	0.219 [-0.029 to 0.466]	–	0.081 [-0.249,0.411]
N01 (anesthetics)	-0.038	-0.037 [-0.138 to 0.064]	0.015	0.138 [0.014–0.262]
N02 (analgesics)	–	-0.022 [-0.085 to 0.040]	0.089	0.116 [0.039–0.193]
N03 (antiepileptics)	–	-0.030 [-0.183 to 0.124]	–	0.019 [-0.172,0.210]
N04 (antiparkinson)	0.120	0.196 [-0.100 to 0.492]	-0.153	-0.521 [-1.099,0.056]
N05 (psycholeptics)	–	-0.028 [-0.130 to 0.073]	–	0.008 [-0.124,0.140]
N06 (psychoanaleptics)	-0.138	-0.225 [-0.386 to -0.064]	0.084	0.138 [-0.052,0.328]
N07 (other nervous)	-0.049	-0.101 [-0.272 to 0.069]	–	0.052 [-0.163,0.266]
P01 (antiprotozoals)	–	-0.022 [-1.209 to 1.166]	–	0.740 [-0.443,1.923]
P02 (anthelmintics)	-0.692	–	–	-0.104 [-2.099,1.891]
P03 (ectoparasiticides)	-0.067	-0.098 [-0.250 to 0.054]	–	0.049 [-0.142,0.241]
R01 (nasal)	0.254	0.272 [0.204–0.340]	0.060	0.062 [-0.027,0.152]
R02 (throat)	0.186	0.198 [0.120–0.277]	0.056	0.074 [-0.029,0.177]
R03 (obstructive airway)	0.141	0.158 [0.093–0.223]	0.085	0.108 [0.024–0.192]
R05 (cough, cold)	0.056	0.087 [0.015–0.159]	0.108	0.115 [0.025–0.205]
R06 (antihistamines)	0.165	0.170 [0.108–0.232]	–	-0.019 [-0.095,0.058]
R07 (other respiratory)	–	-0.352 [-2.343 to 1.639]	–	-0.070 [-2.059,1.920]
S01 (eye)	0.264	0.267 [0.185–0.349]	0.037	0.059 [-0.042,0.160]
S02 (ear)	–	0.004 [-0.090 to 0.098]	–	-0.097 [-0.217,0.023]
S03 (eye/ear)	-0.031	-0.065 [-0.154 to 0.023]	0.038	0.073 [-0.039,0.185]
V01 (allergens)	0.012	0.176 [-0.331 to 0.683]	–	0.021 [-0.683,0.724]
V03 (other therapeutic)	1.252	1.259 [1.200–1.318]	0.720	0.734 [0.661–0.807]
V04 (diagnostic)	–	0.001 [-0.143 to 0.145]	–	-0.038 [-0.229,0.154]
V06 (nutrients)	–	0.009 [-0.151 to 0.168]	–	-0.118 [-0.343,0.108]
V07 (other non-therapeutic)	-0.109	-0.128 [-0.218 to -0.037]	–	0.013 [-0.101,0.126]
V08 (contrast media)	–	0.093 [-0.050 to 0.236]	-0.078	-0.116 [-0.312,0.080]
V09 (diagnostic radiopharmaceuticals)	–	0.124 [-0.369 to 0.618]	-0.112	-0.516 [-1.416,0.384]
V10 (therapeutic radiopharmaceuticals)	–	–	–	–
V20 (surgical dressings)	-0.032	-0.087 [-0.155 to -0.018]	–	-0.003 [-0.086,0.080]

– : omitted variable

eTable 6. Coefficients of the lasso and conventional logistic regression models for prescription of Kampo formulations (goreisan, kikyoto)

Variable	Goreisan		Kikyoto	
	Lasso	Conventional logistic [95% confidence interval]	Lasso	Conventional logistic [95% confidence interval]
Basic characteristics				
Age	-0.015	-0.018 [-0.021 to -0.014]	-0.007	-0.010 [-0.014,-0.006]
Male	-0.207	-0.258 [-0.354 to -0.161]	-0.014	-0.080 [-0.183,0.022]
Employee	–	0.085 [-0.024 to 0.195]	–	0.070 [-0.048,0.188]
Number of family members	–	0.008 [-0.026 to 0.043]	–	0.002 [-0.034,0.037]
Medical cost	-0.001	-0.001 [-0.002 to 0.000]	-0.001	-0.002 [-0.004,-0.001]
Diagnosis, International Statistical Classification of Diseases and Related Health Problems 10th Revision chapter				
I (infectious, parasitic)	0.156	0.165 [0.069–0.260]	0.142	0.191 [0.090–0.293]
II (neoplasms)	–	0.017 [-0.096 to 0.130]	–	0.065 [-0.059,0.190]
III (blood, immune)	–	-0.044 [-0.177 to 0.088]	–	-0.092 [-0.247,0.064]
IV (endocrine, metabolic)	0.031	0.106 [0.009–0.202]	–	-0.008 [-0.118,0.102]
V (mental)	0.347	0.421 [0.287–0.554]	0.028	0.142 [-0.024,0.307]
VI (nervous)	0.175	0.198 [0.091–0.305]	–	-0.009 [-0.133,0.115]
VII (eye)	0.004	0.009 [-0.094 to 0.112]	0.081	0.100 [-0.009,0.210]
VIII (ear)	0.209	0.204 [0.090–0.318]	–	0.005 [-0.125,0.135]
IX (circulatory)	0.070	0.190 [0.066–0.313]	–	0.093 [-0.050,0.235]
X (respiratory)	0.079	0.102 [-0.014 to 0.219]	0.194	0.190 [0.056–0.323]
XI (digestive)	0.078	0.115 [0.021–0.209]	0.076	0.149 [0.050–0.247]
XII (skin)	–	0.003 [-0.105 to 0.112]	0.122	0.195 [0.082–0.308]
XIII (musculoskeletal)	0.130	0.178 [0.080–0.277]	0.119	0.154 [0.048–0.260]
XIV (genitourinary)	0.211	0.232 [0.133–0.330]	0.152	0.198 [0.090–0.307]
XV (pregnancy, birth, puerperium)	0.131	0.177 [-0.043 to 0.396]	0.259	0.329 [0.092–0.566]
XVI (perinatal)	0.171	0.323 [-0.157 to 0.804]	-0.253	-0.684 [-1.454,0.085]
XVII (congenital)	–	-0.129 [-0.400 to 0.143]	–	-0.010 [-0.302,0.281]
XVIII (symptoms, signs, findings)	0.381	0.376 [0.292–0.459]	0.112	0.140 [0.051–0.228]
XIX (injury, poisoning)	0.019	0.074 [-0.028 to 0.176]	–	0.033 [-0.079,0.145]
XX (external cause)	–	–	–	–
XXI (others)	–	–	–	–
XXII (special purpose)	–	-0.045 [-0.319 to 0.230]	–	-0.026 [-0.329,0.278]
Medical service				
Consultation, hospitalization	–	-0.021 [-0.286 to 0.243]	0.123	0.239 [-0.045,0.524]
Disease management fee	0.013	0.035 [-0.051 to 0.121]	–	-0.009 [-0.098,0.080]
Home medical care	-0.140	-0.257 [-0.565 to 0.050]	–	0.188 [-0.119,0.495]
Examination	0.166	0.201 [0.076–0.326]	–	-0.058 [-0.178,0.063]
Imaging	–	-0.039 [-0.130 to 0.051]	–	-0.051 [-0.147,0.045]
Prescription	0.030	0.101 [-0.123 to 0.325]	0.037	0.059 [-0.186,0.304]
Injection	–	-0.017 [-0.133 to 0.099]	-0.042	-0.108 [-0.236,0.020]
Rehabilitation	–	-0.026 [-0.207 to 0.154]	0.013	0.132 [-0.065,0.330]
Psychiatric treatment	-0.063	-0.279 [-0.457 to -0.100]	–	0.011 [-0.213,0.235]
Procedure	0.017	0.054 [-0.038 to 0.147]	0.025	0.052 [-0.047,0.152]
Operation	-0.179	-0.190 [-0.357 to -0.023]	-0.078	-0.203 [-0.380,-0.026]
Anesthesia	–	0.076 [-0.121 to 0.273]	–	0.138 [-0.077,0.353]
Radiotherapy	-0.268	-0.836 [-2.042 to 0.370]	-0.282	-1.131 [-3.146,0.883]
Pathology	-0.046	-0.113 [-0.233 to 0.007]	–	-0.064 [-0.197,0.069]

– : omitted variable

eTable 6 (continued). Coefficients of the lasso and conventional logistic regression models for prescription of Kampo formulations (goreisan, kikyoto)

Variable	Goreisan		Kikyoto	
	Lasso	Conventional logistic [95% confidence interval]	Lasso	Conventional logistic [95% confidence interval]
Prescription, Anatomical Therapeutic Chemical Classification System code				
A01 (stomatological)	–	-0.003 [-0.122 to 0.115]	0.231	0.235 [0.124–0.347]
A02 (acid related)	–	-0.029 [-0.124 to 0.066]	–	-0.088 [-0.188 to 0.011]
A03 (functional gastrointestinal)	0.034	0.047 [-0.052 to 0.146]	–	-0.100 [-0.211 to 0.011]
A04 (antiemetics, antinauseants)	–	0.838 [-0.177 to 1.853]	–	-0.068 [-1.475 to 1.339]
A05 (bile, liver)	-0.161	-0.297 [-0.586 to -0.009]	-0.173	-0.324 [-0.648 to 0.000]
A06 (constipation)	–	-0.001 [-0.141 to 0.140]	-0.026	-0.072 [-0.233 to 0.089]
A07 (antidiarrheals)	0.169	0.181 [0.080–0.282]	0.082	0.099 [-0.009 to 0.207]
A08 (antiobesity)	–	–	–	–
A09 (digestives)	0.046	0.110 [-0.101 to 0.320]	0.019	0.115 [-0.121 to 0.352]
A10 (diabetes)	–	-0.094 [-0.353 to 0.166]	-0.118	-0.328 [-0.635 to -0.022]
A11 (vitamins)	–	-0.038 [-0.166 to 0.090]	-0.004	-0.090 [-0.234 to 0.055]
A12 (mineral supplements)	–	0.242 [-0.187 to 0.672]	–	0.171 [-0.365 to 0.707]
A14 (anabolic)	–	–	–	–
A15 (appetite stimulants)	–	0.384 [-1.639 to 2.407]	–	0.497 [-1.508 to 2.502]
A16 (other alimentary/metabolism)	–	0.140 [-0.257 to 0.536]	0.363	0.518 [0.145–0.891]
B01 (antithrombotic)	-0.176	-0.262 [-0.479 to -0.045]	-0.001	-0.050 [-0.291 to 0.191]
B02 (antihemorrhagics)	0.032	0.079 [-0.008 to 0.166]	0.288	0.292 [0.203–0.380]
B03 (antianemic)	–	-0.006 [-0.139 to 0.127]	–	0.005 [-0.154 to 0.163]
B05 (blood substitutes, perfusion)	–	0.019 [-0.088 to 0.126]	–	0.013 [-0.101 to 0.127]
B06 (other hematological)	–	–	–	–
C01 (cardiac)	0.071	0.142 [-0.011 to 0.296]	–	-0.021 [-0.208 to 0.166]
C02 (antihypertensives)	–	-0.145 [-0.709 to 0.419]	–	0.129 [-0.482 to 0.739]
C03 (diuretics)	0.161	0.375 [0.069–0.681]	–	-0.072 [-0.514 to 0.370]
C04 (peripheral vasodilators)	–	-0.111 [-0.379 to 0.156]	–	-0.139 [-0.473 to 0.194]
C05 (vasoprotectives)	0.015	0.043 [-0.070 to 0.156]	0.055	0.101 [-0.018 to 0.220]
C07 (beta blocking agents)	0.063	0.203 [-0.047 to 0.454]	-0.136	-0.313 [-0.681 to 0.055]
C08 (calcium channel blockers)	–	-0.092 [-0.281 to 0.097]	-0.027	-0.132 [-0.355 to 0.090]
C09 (renin-angiotensin system)	–	-0.116 [-0.305 to 0.073]	-0.074	-0.188 [-0.408 to 0.032]
C10 (lipid modifying)	-0.094	-0.205 [-0.375 to -0.036]	–	0.169 [-0.008 to 0.346]
D01 (antifungals)	–	0.009 [-0.136 to 0.154]	–	-0.061 [-0.219 to 0.097]
D02 (emollients, protectives)	–	-0.072 [-0.216 to 0.072]	–	-0.057 [-0.210 to 0.096]
D03 (wounds and ulcers)	-0.054	-0.216 [-0.517 to 0.086]	–	-0.152 [-0.469 to 0.166]
D04 (antipruritics)	0.088	0.187 [-0.036 to 0.410]	0.049	0.158 [-0.086 to 0.402]
D05 (antipsoriatics)	–	-0.232 [-0.733 to 0.269]	–	-0.075 [-0.561 to 0.411]
D06 (dermatological antibiotics)	–	-0.043 [-0.182 to 0.096]	–	-0.014 [-0.161 to 0.133]
D07 (dermatological steroids)	0.018	0.044 [-0.070 to 0.157]	–	-0.079 [-0.199 to 0.042]
D08 (antiseptics, disinfectants)	–	-0.043 [-0.256 to 0.170]	-0.040	-0.144 [-0.386 to 0.097]
D09 (medicated dressings)	–	–	–	–
D10 (antiacne)	–	-0.025 [-0.185 to 0.135]	–	-0.038 [-0.207 to 0.131]
D11 (other dermatological)	–	0.060 [-0.128 to 0.247]	–	-0.018 [-0.222 to 0.186]
G01 (gynecological antiinfectives)	–	-0.101 [-0.294 to 0.092]	-0.097	-0.245 [-0.472 to -0.017]
G02 (other gynecological)	0.033	0.151 [-0.139 to 0.441]	–	-0.059 [-0.381 to 0.264]
G03 (sex hormones)	0.109	0.112 [-0.043 to 0.267]	–	-0.036 [-0.225 to 0.154]
G04 (urologicals)	–	0.014 [-0.256 to 0.283]	–	0.117 [-0.175 to 0.409]
H01 (pituitary, hypothalamic)	–	-0.169 [-0.531 to 0.193]	–	0.081 [-0.298 to 0.460]
H02 (systemic steroids)	–	0.025 [-0.075 to 0.126]	–	-0.060 [-0.166 to 0.047]
H03 (thyroid)	-0.039	-0.267 [-0.573 to 0.039]	–	0.130 [-0.178 to 0.439]
H04 (pancreatic)	–	0.037 [-0.528 to 0.603]	-0.153	-0.546 [-1.363 to 0.270]
H05 (calcium homeostasis)	–	-0.223 [-1.441 to 0.996]	–	0.255 [-1.243 to 1.754]

– : omitted variable

eTable 6 (continued). Coefficients of the lasso and conventional logistic regression models for prescription of Kampo formulations (goreisan, kikyoto)

Variable	Goreisan		Kikyoto	
	Lasso	Conventional logistic [95% confidence interval]	Lasso	Conventional logistic [95% confidence interval]
Prescription, Anatomical Therapeutic Chemical Classification System code				
J01 (antibacterials)	–	-0.044 [-0.137 to 0.049]	0.064	0.072 [-0.028 to 0.172]
J02 (antimycotics)	-0.064	-0.503 [-1.500 to 0.494]	0.217	0.614 [-0.061 to 1.289]
J04 (antimycobacterials)	–	-0.400 [-2.380 to 1.580]	–	-0.128 [-2.110 to 1.854]
J05 (antivirals)	–	-0.003 [-0.107 to 0.101]	-0.029	-0.105 [-0.215 to 0.006]
J06 (sera, immunoglobulins)	–	-0.166 [-0.922 to 0.590]	–	-0.025 [-0.780 to 0.731]
J07 (vaccines)	–	0.149 [-0.753 to 1.051]	–	-0.584 [-1.985 to 0.817]
L01 (antineoplastic)	–	-0.437 [-1.134 to 0.261]	–	0.183 [-0.457 to 0.822]
L02 (endocrine)	–	0.234 [-0.159 to 0.627]	–	-0.047 [-0.564 to 0.471]
L03 (immunostimulants)	–	-0.334 [-1.513 to 0.846]	-0.061	-0.826 [-2.970 to 1.319]
L04 (immunosuppressants)	-0.220	-0.447 [-0.957 to 0.063]	–	0.143 [-0.348 to 0.633]
M01 (antiinflammatory, antirheumatic)	-0.006	-0.084 [-0.172 to 0.005]	–	0.024 [-0.068 to 0.116]
M02 (joint/muscular topical)	0.108	0.154 [0.045–0.263]	–	0.040 [-0.081 to 0.160]
M03 (muscle relaxants)	–	-0.065 [-0.215 to 0.084]	-0.099	-0.186 [-0.363 to -0.009]
M04 (antigout)	-0.179	-0.338 [-0.594 to -0.081]	–	-0.057 [-0.306 to 0.192]
M05 (bone)	–	0.015 [-0.540 to 0.570]	-0.022	-0.354 [-1.124 to 0.416]
M09 (other musculo-skeletal)	–	-0.039 [-0.386 to 0.309]	–	-0.047 [-0.438 to 0.344]
N01 (anesthetics)	-0.005	-0.067 [-0.200 to 0.065]	0.016	0.158 [0.020–0.296]
N02 (analgesics)	–	0.001 [-0.084 to 0.087]	0.011	0.057 [-0.032 to 0.147]
N03 (antiepileptics)	0.123	0.185 [0.021–0.348]	–	-0.070 [-0.290 to 0.150]
N04 (antiparkinson)	-0.122	-0.310 [-0.736 to 0.116]	–	-0.092 [-0.600 to 0.415]
N05 (psycholeptics)	–	-0.001 [-0.126 to 0.124]	–	-0.054 [-0.199 to 0.091]
N06 (psychoanaleptics)	0.005	0.125 [-0.047 to 0.298]	–	-0.129 [-0.351 to 0.093]
N07 (other nervous)	0.211	0.222 [0.051–0.394]	–	0.072 [-0.156 to 0.300]
P01 (antiprotozoals)	–	0.207 [-1.226 to 1.640]	-0.174	–
P02 (anthelmintics)	-0.009	–	0.617	1.054 [-0.404 to 2.513]
P03 (ectoparasiticides)	–	-0.067 [-0.258 to 0.123]	–	-0.068 [-0.279 to 0.143]
R01 (nasal)	0.067	0.085 [-0.011 to 0.182]	0.014	0.028 [-0.073 to 0.129]
R02 (throat)	-0.002	-0.080 [-0.199 to 0.038]	0.185	0.200 [0.090–0.309]
R03 (obstructive airway)	–	0.022 [-0.072 to 0.115]	–	-0.043 [-0.140 to 0.053]
R05 (cough, cold)	–	-0.009 [-0.108 to 0.090]	0.137	0.146 [0.040–0.253]
R06 (antihistamines)	0.027	0.033 [-0.052 to 0.119]	0.033	0.048 [-0.041 to 0.137]
R07 (other respiratory)	–	-0.099 [-2.095 to 1.896]	0.296	1.034 [-0.401 to 2.468]
S01 (eye)	0.095	0.099 [-0.012 to 0.210]	0.063	0.074 [-0.044 to 0.191]
S02 (ear)	–	-0.027 [-0.155 to 0.102]	–	0.024 [-0.107 to 0.155]
S03 (eye/ear)	–	0.009 [-0.113 to 0.130]	0.006	-0.004 [-0.129 to 0.122]
V01 (allergens)	–	-0.126 [-0.940 to 0.688]	0.688	0.867 [0.326–1.409]
V03 (other therapeutic)	0.988	0.983 [0.903–1.064]	1.051	1.053 [0.968–1.138]
V04 (diagnostic)	–	0.123 [-0.062 to 0.309]	–	0.044 [-0.163 to 0.250]
V06 (nutrients)	–	-0.093 [-0.304 to 0.117]	0.058	0.241 [0.014–0.467]
V07 (other non-therapeutic)	-0.017	-0.106 [-0.229 to 0.018]	–	0.000 [-0.126 to 0.126]
V08 (contrast media)	–	-0.035 [-0.219 to 0.148]	-0.005	-0.026 [-0.238 to 0.185]
V09 (diagnostic radiopharmaceuticals)	–	-0.149 [-0.774 to 0.476]	–	0.019 [-0.758 to 0.795]
V10 (therapeutic radiopharmaceuticals)	–	–	–	–
V20 (surgical dressings)	–	–	–	–

– : omitted variable

eTable 7. Coefficients of the lasso and conventional logistic regression models for prescription of Kampo formulations (maobushisaishinto, shakuyakukanzoto)

Variable	Maobushisaishinto		Shakuyakukanzoto	
	Lasso	Conventional logistic [95% confidence interval]	Lasso	Conventional logistic [95% confidence interval]
Basic characteristics				
Age	-0.002	-0.005 [-0.009 to -0.001]	0.034	0.036 [0.031–0.042]
Male	–	-0.076 [-0.179 to 0.027]	-0.101	-0.211 [-0.339,-0.084]
Employee	0.058	0.191 [0.071–0.311]	0.120	0.243 [0.104–0.383]
Number of family members	–	0.033 [-0.001 to 0.067]	-0.009	-0.003 [-0.045,0.040]
Medical cost	–	0.000 [0.000–0.001]	–	0.000 [-0.001,0.000]
Diagnosis, International Statistical Classification of Diseases and Related Health Problems 10th Revision chapter				
I (infectious, parasitic)	0.012	0.019 [-0.087 to 0.125]	0.052	0.079 [-0.038,0.196]
II (neoplasms)	-0.048	-0.084 [-0.221 to 0.052]	–	0.017 [-0.115,0.148]
III (blood, immune)	–	-0.021 [-0.183 to 0.141]	–	0.001 [-0.149,0.152]
IV (endocrine, metabolic)	–	-0.014 [-0.127 to 0.099]	0.095	0.101 [-0.016,0.219]
V (mental)	0.042	0.070 [-0.102 to 0.242]	0.072	0.144 [-0.012,0.301]
VI (nervous)	0.087	0.149 [0.026–0.271]	0.347	0.326 [0.206–0.446]
VII (eye)	0.023	0.067 [-0.047 to 0.182]	0.020	0.050 [-0.080,0.180]
VIII (ear)	–	0.006 [-0.125 to 0.138]	–	0.044 [-0.104,0.192]
IX (circulatory)	–	0.098 [-0.047 to 0.242]	0.263	0.247 [0.113–0.380]
X (respiratory)	0.229	0.255 [0.125–0.385]	–	-0.011 [-0.151,0.128]
XI (digestive)	0.015	0.049 [-0.052 to 0.149]	0.005	0.038 [-0.077,0.153]
XII (skin)	0.053	0.072 [-0.046 to 0.190]	0.201	0.191 [0.065–0.317]
XIII (musculoskeletal)	0.113	0.150 [0.042–0.258]	0.502	0.499 [0.379–0.619]
XIV (genitourinary)	–	0.016 [-0.100 to 0.131]	0.119	0.145 [0.025–0.264]
XV (pregnancy, birth, puerperium)	-0.023	-0.398 [-0.729 to -0.067]	–	-0.068 [-0.462,0.325]
XVI (perinatal)	–	0.066 [-0.633 to 0.766]	-0.183	-0.755 [-2.178,0.668]
XVII (congenital)	0.076	0.197 [-0.077 to 0.472]	0.156	0.228 [-0.030,0.487]
XVIII (symptoms, signs, findings)	0.050	0.091 [0.000–0.182]	0.407	0.438 [0.336–0.540]
XIX (injury, poisoning)	–	0.049 [-0.064 to 0.163]	–	-0.018 [-0.136,0.099]
XX (external cause)	–	–	–	–
XXI (others)	–	–	–	–
XXII (special purpose)	–	0.021 [-0.287 to 0.329]	–	-0.072 [-0.338,0.195]
Medical service				
Consultation, hospitalization	–	0.017 [-0.258 to 0.292]	0.028	0.279 [-0.121,0.678]
Disease management fee	0.051	0.085 [-0.005 to 0.176]	0.108	0.139 [0.022–0.256]
Home medical care	0.014	0.162 [-0.122 to 0.446]	0.222	0.278 [0.060–0.496]
Examination	–	-0.121 [-0.238 to -0.005]	–	-0.123 [-0.270,0.025]
Imaging	–	-0.071 [-0.169 to 0.027]	-0.054	-0.140 [-0.251,-0.029]
Prescription	0.000	0.074 [-0.174 to 0.323]	0.240	0.292 [-0.043,0.628]
Injection	–	-0.027 [-0.158 to 0.103]	0.075	0.160 [0.026–0.293]
Rehabilitation	–	0.093 [-0.105 to 0.291]	-0.038	-0.103 [-0.279,0.073]
Psychiatric treatment	0.038	0.096 [-0.129 to 0.322]	-0.209	-0.345 [-0.569,-0.122]
Procedure	0.079	0.082 [-0.019 to 0.183]	-0.085	-0.158 [-0.269,-0.047]
Operation	-0.097	-0.140 [-0.325 to 0.045]	-0.116	-0.109 [-0.290,0.073]
Anesthesia	-0.097	-0.104 [-0.342 to 0.134]	–	0.074 [-0.122,0.269]
Radiotherapy	-0.099	-0.774 [-2.260 to 0.713]	0.181	0.413 [-0.239,1.064]
Pathology	-0.077	-0.087 [-0.232 to 0.058]	0.000	-0.060 [-0.202,0.082]

– : omitted variable

eTable 7 (continued). Coefficients of the lasso and conventional logistic regression models for prescription of Kampo formulations (maobushisaishinto, shakuyakukanzoto)

Variable	Maobushisaishinto		Shakuyakukanzoto	
	Lasso	Conventional logistic [95% confidence interval]	Lasso	Conventional logistic [95% confidence interval]
Prescription, Anatomical Therapeutic Chemical Classification System code				
A01 (stomatological)	0.061	0.083 [-0.039 to 0.205]	–	-0.010 [-0.157 to 0.136]
A02 (acid related)	0.004	0.027 [-0.074 to 0.129]	–	-0.033 [-0.150 to 0.084]
A03 (functional gastrointestinal)	–	-0.027 [-0.142 to 0.088]	–	-0.041 [-0.164 to 0.082]
A04 (antiemetics, antinauseants)	–	-0.087 [-1.395 to 1.221]	-0.318	-0.500 [-1.384 to 0.384]
A05 (bile, liver)	-0.067	-0.249 [-0.560 to 0.062]	0.005	0.057 [-0.192 to 0.305]
A06 (constipation)	-0.035	-0.064 [-0.235 to 0.106]	-0.014	-0.049 [-0.209 to 0.111]
A07 (antidiarrheals)	0.092	0.105 [-0.007 to 0.218]	0.067	0.109 [-0.015 to 0.234]
A08 (antiobesity)	–	–	-0.360	–
A09 (digestives)	–	0.091 [-0.159 to 0.342]	–	0.062 [-0.202 to 0.326]
A10 (diabetes)	-0.116	-0.338 [-0.623 to -0.053]	0.273	0.302 [0.116–0.488]
A11 (vitamins)	–	0.013 [-0.135 to 0.160]	0.008	0.016 [-0.129 to 0.162]
A12 (mineral supplements)	–	0.187 [-0.322 to 0.697]	0.312	0.356 [0.000–0.711]
A14 (anabolic)	–	–	1.347	1.727 [-0.533 to 3.987]
A15 (appetite stimulants)	–	–	1.040	1.294 [-0.222 to 2.809]
A16 (other alimentary/metabolism)	–	0.080 [-0.387 to 0.547]	0.207	0.243 [-0.153 to 0.640]
B01 (antithrombotic)	-0.105	-0.220 [-0.473 to 0.034]	0.064	0.149 [-0.030 to 0.328]
B02 (antihemorrhagics)	0.040	0.061 [-0.031 to 0.154]	–	0.013 [-0.095 to 0.121]
B03 (antianemic)	-0.009	-0.095 [-0.261 to 0.070]	0.010	0.067 [-0.078 to 0.212]
B05 (blood substitutes, perfusion)	–	0.042 [-0.073 to 0.158]	-0.024	-0.086 [-0.216 to 0.045]
B06 (other hematological)	–	–	–	–
C01 (cardiac)	-0.145	-0.191 [-0.392 to 0.010]	-0.010	-0.074 [-0.250 to 0.103]
C02 (antihypertensives)	0.016	0.268 [-0.263 to 0.798]	0.160	0.231 [-0.154 to 0.615]
C03 (diuretics)	–	-0.093 [-0.500 to 0.315]	0.428	0.454 [0.219–0.688]
C04 (peripheral vasodilators)	-0.061	-0.253 [-0.620 to 0.113]	–	-0.016 [-0.307 to 0.275]
C05 (vasoprotectives)	–	0.018 [-0.108 to 0.144]	0.053	0.082 [-0.051 to 0.214]
C07 (beta blocking agents)	-0.003	-0.201 [-0.530 to 0.128]	0.003	0.052 [-0.170 to 0.273]
C08 (calcium channel blockers)	–	0.030 [-0.172 to 0.232]	0.013	0.017 [-0.138 to 0.173]
C09 (renin-angiotensin system)	–	-0.068 [-0.269 to 0.134]	0.022	0.020 [-0.135 to 0.176]
C10 (lipid modifying)	0.024	0.145 [-0.026 to 0.317]	0.044	0.039 [-0.103 to 0.182]
D01 (antifungals)	–	0.013 [-0.148 to 0.175]	0.123	0.152 [-0.011 to 0.316]
D02 (emollients, protectives)	-0.015	-0.155 [-0.316 to 0.005]	–	-0.087 [-0.254 to 0.080]
D03 (wounds and ulcers)	–	-0.124 [-0.446 to 0.199]	–	-0.090 [-0.413 to 0.232]
D04 (antipruritics)	-0.185	-0.357 [-0.674 to -0.039]	–	0.046 [-0.211 to 0.303]
D05 (antipsoriatics)	0.199	0.345 [-0.053 to 0.742]	–	0.052 [-0.416 to 0.520]
D06 (dermatological antibiotics)	0.010	0.071 [-0.079 to 0.222]	0.084	0.152 [-0.005 to 0.309]
D07 (dermatological steroids)	0.056	0.086 [-0.039 to 0.211]	–	0.016 [-0.116 to 0.148]
D08 (antiseptics, disinfectants)	–	0.088 [-0.145 to 0.321]	–	0.044 [-0.169 to 0.257]
D09 (medicated dressings)	–	–	–	–
D10 (antiacne)	–	-0.167 [-0.350 to 0.017]	0.043	0.122 [-0.081 to 0.325]
D11 (other dermatological)	0.043	0.123 [-0.076 to 0.323]	–	-0.045 [-0.276 to 0.186]
G01 (gynecological antiinfectives)	–	-0.065 [-0.313 to 0.182]	–	-0.011 [-0.284 to 0.262]
G02 (other gynecological)	0.072	0.456 [0.059–0.854]	-0.008	-0.104 [-0.629 to 0.421]
G03 (sex hormones)	–	-0.011 [-0.219 to 0.198]	0.210	0.227 [0.033–0.421]
G04 (urologicals)	0.041	0.164 [-0.133 to 0.460]	0.067	0.123 [-0.135 to 0.381]
H01 (pituitary, hypothalamic)	–	0.196 [-0.263 to 0.656]	–	-0.028 [-0.586 to 0.529]
H02 (systemic steroids)	0.029	0.066 [-0.041 to 0.173]	–	0.009 [-0.112 to 0.129]
H03 (thyroid)	–	-0.073 [-0.423 to 0.278]	-0.004	-0.150 [-0.464 to 0.164]
H04 (pancreatic)	–	-0.029 [-0.703 to 0.646]	-0.096	-0.257 [-0.832 to 0.318]
H05 (calcium homeostasis)	-0.804	–	0.948	0.907 [0.391–1.423]

– : omitted variable

eTable 7 (continued). Coefficients of the lasso and conventional logistic regression models for prescription of Kampo formulations (maobushisaishinto, shakuyakukanzoto)

Variable	Maobushisaishinto		Shakuyakukanzoto	
	Lasso	Conventional logistic [95% confidence interval]	Lasso	Conventional logistic [95% confidence interval]
Prescription, Anatomical Therapeutic Chemical Classification System code				
J01 (antibacterials)	0.053	0.079 [-0.022 to 0.180]	-0.006	-0.108 [-0.222 to 0.006]
J02 (antimycotics)	0.168	0.541 [-0.144 to 1.225]	0.192	0.405 [-0.304 to 1.113]
J04 (antimycobacterials)	-0.011	–	-0.049	-0.682 [-2.727 to 1.363]
J05 (antivirals)	–	0.009 [-0.103 to 0.121]	-0.076	-0.138 [-0.270 to -0.005]
J06 (sera, immunoglobulins)	0.550	0.723 [0.209–1.238]	–	-0.038 [-0.785 to 0.708]
J07 (vaccines)	–	-0.712 [-2.113 to 0.688]	–	-0.426 [-1.590 to 0.738]
L01 (antineoplastic)	–	0.082 [-0.530 to 0.694]	-0.077	-0.267 [-0.851 to 0.317]
L02 (endocrine)	–	-0.134 [-0.707 to 0.439]	0.014	0.093 [-0.312 to 0.497]
L03 (immunostimulants)	–	-0.548 [-2.202 to 1.106]	0.424	0.716 [-0.064 to 1.495]
L04 (immunosuppressants)	–	-0.036 [-0.509 to 0.438]	-0.385	-0.634 [-1.102 to -0.166]
M01 (antiinflammatory, antirheumatic)	–	-0.018 [-0.112 to 0.076]	0.063	0.125 [0.014–0.235]
M02 (joint/muscular topical)	–	0.043 [-0.079 to 0.165]	0.245	0.273 [0.154–0.391]
M03 (muscle relaxants)	-0.047	-0.124 [-0.301 to 0.053]	–	-0.035 [-0.189 to 0.118]
M04 (antigout)	–	0.037 [-0.189 to 0.262]	–	-0.019 [-0.208 to 0.169]
M05 (bone)	–	-0.267 [-0.990 to 0.456]	–	0.051 [-0.377 to 0.478]
M09 (other musculo-skeletal)	–	-0.205 [-0.615 to 0.205]	0.424	0.412 [0.166–0.657]
N01 (anesthetics)	-0.058	-0.099 [-0.248 to 0.049]	–	-0.007 [-0.153 to 0.140]
N02 (analgesics)	–	-0.067 [-0.158 to 0.024]	-0.004	-0.061 [-0.166 to 0.044]
N03 (antiepileptics)	–	-0.058 [-0.266 to 0.150]	0.235	0.282 [0.116–0.448]
N04 (antiparkinson)	–	-0.026 [-0.519 to 0.467]	0.187	0.328 [-0.075 to 0.730]
N05 (psycholeptics)	–	-0.118 [-0.267 to 0.031]	–	0.044 [-0.096 to 0.184]
N06 (psychoanaleptics)	–	0.032 [-0.178 to 0.242]	-0.110	-0.188 [-0.408 to 0.032]
N07 (other nervous)	–	-0.044 [-0.286 to 0.199]	–	-0.067 [-0.286 to 0.151]
P01 (antiprotozoals)	-0.308	–	0.061	0.363 [-0.744 to 1.471]
P02 (anthelmintics)	–	0.742 [-1.263 to 2.747]	–	–
P03 (ectoparasiticides)	–	-0.020 [-0.244 to 0.205]	–	0.045 [-0.163 to 0.252]
R01 (nasal)	0.079	0.070 [-0.032 to 0.172]	0.012	0.054 [-0.065 to 0.173]
R02 (throat)	0.137	0.162 [0.045–0.279]	0.143	0.182 [0.045–0.319]
R03 (obstructive airway)	0.108	0.122 [0.025–0.218]	0.127	0.162 [0.048–0.276]
R05 (cough, cold)	–	-0.042 [-0.148 to 0.063]	–	0.056 [-0.066 to 0.179]
R06 (antihistamines)	0.023	0.018 [-0.073 to 0.108]	0.077	0.093 [-0.011 to 0.197]
R07 (other respiratory)	–	–	–	0.020 [-1.998 to 2.037]
S01 (eye)	0.199	0.191 [0.070–0.312]	–	-0.007 [-0.145 to 0.131]
S02 (ear)	0.091	0.106 [-0.026 to 0.239]	–	0.048 [-0.126 to 0.222]
S03 (eye/ear)	0.047	0.075 [-0.052 to 0.201]	–	-0.037 [-0.197 to 0.124]
V01 (allergens)	–	0.200 [-0.508 to 0.908]	–	0.164 [-0.733 to 1.061]
V03 (other therapeutic)	1.216	1.239 [1.153–1.325]	0.921	0.938 [0.838–1.038]
V04 (diagnostic)	–	0.074 [-0.149 to 0.298]	–	-0.005 [-0.222 to 0.213]
V06 (nutrients)	–	0.077 [-0.174 to 0.328]	–	-0.059 [-0.298 to 0.181]
V07 (other non-therapeutic)	–	-0.071 [-0.201 to 0.059]	-0.120	-0.189 [-0.351 to -0.027]
V08 (contrast media)	-0.161	-0.199 [-0.432 to 0.033]	-0.081	-0.114 [-0.307 to 0.079]
V09 (diagnostic radiopharmaceuticals)	–	0.413 [-0.250 to 1.076]	0.148	0.273 [-0.172 to 0.717]
V10 (therapeutic radiopharmaceuticals)	–	–	–	–
V20 (surgical dressings)	–	–	–	–

– : omitted variable

eTable 8. Coefficients of the lasso and conventional logistic regression models for prescription of Kampo formulations (tokishakuyakusan, kakkontokasenkyushin'i)

Variable	Tokishakuyakusan		Kakkontokasenkyushin'i	
	Lasso	Conventional logistic [95% confidence interval]	Lasso	Conventional logistic [95% confidence interval]
Basic characteristics				
Age	-0.015	-0.018 [-0.023 to -0.013]	0.000	-0.005 [-0.010 to 0.000]
Male	-2.608	-2.731 [-2.978 to -2.485]	-0.007	-0.059 [-0.184 to 0.067]
Employee	-0.200	-0.351 [-0.502 to -0.201]	-0.028	-0.011 [-0.153 to 0.130]
Number of family members	-0.075	-0.136 [-0.194 to -0.079]	0.013	0.035 [-0.007 to 0.077]
Medical cost	0.000	-0.001 [-0.002 to 0.001]	-0.001	-0.002 [-0.003 to 0.000]
Diagnosis, International Statistical Classification of Diseases and Related Health Problems 10th Revision chapter				
I (infectious, parasitic)	-0.013	-0.098 [-0.218 to 0.021]	0.044	0.080 [-0.043 to 0.202]
II (neoplasms)	0.113	0.141 [0.027–0.256]	0.020	0.128 [-0.019 to 0.276]
III (blood, immune)	0.216	0.263 [0.131–0.395]	-0.059	-0.229 [-0.422 to -0.036]
IV (endocrine, metabolic)	0.292	0.329 [0.219–0.439]	-0.026	-0.108 [-0.243 to 0.027]
V (mental)	0.326	0.374 [0.216–0.533]	–	-0.116 [-0.326 to 0.094]
VI (nervous)	0.209	0.246 [0.118–0.374]	0.048	0.129 [-0.016 to 0.274]
VII (eye)	0.022	0.043 [-0.085 to 0.171]	–	-0.037 [-0.172 to 0.099]
VIII (ear)	0.005	0.046 [-0.099 to 0.192]	–	0.035 [-0.108 to 0.179]
IX (circulatory)	–	0.037 [-0.117 to 0.191]	–	0.218 [0.053–0.384]
X (respiratory)	–	-0.084 [-0.226 to 0.057]	0.280	0.372 [0.200–0.544]
XI (digestive)	0.009	0.070 [-0.049 to 0.189]	0.026	0.120 [0.001–0.238]
XII (skin)	0.012	0.073 [-0.055 to 0.201]	0.108	0.152 [0.017–0.288]
XIII (musculoskeletal)	0.113	0.193 [0.074–0.312]	–	0.030 [-0.100 to 0.159]
XIV (genitourinary)	0.746	0.745 [0.630–0.861]	–	0.028 [-0.105 to 0.160]
XV (pregnancy, birth, puerperium)	–	-0.004 [-0.217 to 0.209]	0.442	0.453 [0.174–0.733]
XVI (perinatal)	-0.643	-0.907 [-1.736 to -0.079]	–	0.075 [-0.562 to 0.712]
XVII (congenital)	-0.071	-0.190 [-0.510 to 0.129]	–	-0.124 [-0.483 to 0.235]
XVIII (symptoms, signs, findings)	0.276	0.302 [0.198–0.406]	0.097	0.144 [0.039–0.250]
XIX (injury, poisoning)	–	0.009 [-0.119 to 0.136]	-0.004	-0.103 [-0.239 to 0.033]
XX (external cause)	–	–	–	–
XXI (others)	–	–	–	–
XXII (special purpose)	-0.148	-0.267 [-0.632 to 0.098]	–	0.079 [-0.257 to 0.416]
Medical service				
Consultation, hospitalization	–	0.076 [-0.328 to 0.480]	–	0.427 [0.088–0.767]
Disease management fee	0.096	0.141 [0.029–0.253]	–	0.001 [-0.106 to 0.108]
Home medical care	0.124	0.429 [0.044–0.814]	–	-0.042 [-0.412 to 0.328]
Examination	–	-0.082 [-0.259 to 0.094]	–	-0.045 [-0.189 to 0.098]
Imaging	–	-0.041 [-0.153 to 0.071]	–	0.042 [-0.071 to 0.156]
Prescription	0.191	0.377 [0.055–0.698]	–	-0.390 [-0.683 to -0.096]
Injection	0.065	0.142 [0.006–0.278]	-0.105	-0.132 [-0.286 to 0.023]
Rehabilitation	–	0.075 [-0.153 to 0.303]	–	0.026 [-0.218 to 0.270]
Psychiatric treatment	–	-0.119 [-0.332 to 0.093]	–	0.199 [-0.079 to 0.477]
Procedure	–	-0.036 [-0.152 to 0.080]	0.083	0.112 [-0.011 to 0.235]
Operation	–	0.003 [-0.190 to 0.196]	–	0.061 [-0.143 to 0.265]
Anesthesia	–	0.097 [-0.150 to 0.344]	-0.039	-0.052 [-0.323 to 0.218]
Radiotherapy	–	-0.121 [-1.201 to 0.958]	–	0.537 [-0.716 to 1.791]
Pathology	–	-0.012 [-0.132 to 0.107]	-0.089	-0.165 [-0.331 to 0.000]

– : omitted variable

eTable 8 (continued). Coefficients of the lasso and conventional logistic regression models for prescription of Kampo formulations (tokishakuyakusan, kakkontokasenkyushin'i)

Variable	Tokishakuyakusan		Kakkontokasenkyushin'i	
	Lasso	Conventional logistic [95% confidence interval]	Lasso	Conventional logistic [95% confidence interval]
Prescription, Anatomical Therapeutic Chemical Classification System code				
A01 (stomatological)	–	0.002 [-0.144 to 0.148]	0.120	0.149 [0.015–0.283]
A02 (acid related)	–	-0.048 [-0.167 to 0.072]	–	-0.038 [-0.158 to 0.082]
A03 (functional gastrointestinal)	–	-0.027 [-0.151 to 0.097]	–	-0.048 [-0.181 to 0.086]
A04 (antiemetics, antinauseants)	-0.647	-0.851 [-2.336 to 0.634]	–	-0.521 [-2.291 to 1.250]
A05 (bile, liver)	–	-0.018 [-0.344 to 0.307]	-0.015	-0.174 [-0.539 to 0.191]
A06 (constipation)	-0.175	-0.210 [-0.387 to -0.033]	-0.106	-0.163 [-0.364 to 0.037]
A07 (antidiarrheals)	–	0.060 [-0.067 to 0.188]	0.094	0.131 [0.003–0.258]
A08 (antiobesity)	-0.456	–	–	–
A09 (digestives)	0.061	0.148 [-0.119 to 0.416]	–	-0.108 [-0.426 to 0.210]
A10 (diabetes)	-0.415	-0.742 [-1.230 to -0.254]	–	-0.093 [-0.437 to 0.251]
A11 (vitamins)	0.049	0.070 [-0.071 to 0.210]	–	-0.054 [-0.228 to 0.120]
A12 (mineral supplements)	-0.090	-0.248 [-0.875 to 0.379]	–	0.208 [-0.391 to 0.806]
A14 (anabolic)	–	–	–	–
A15 (appetite stimulants)	–	–	–	0.775 [-1.237 to 2.787]
A16 (other alimentary/metabolism)	-0.092	-0.288 [-0.773 to 0.198]	–	-0.123 [-0.732 to 0.485]
B01 (antithrombotic)	-0.131	-0.171 [-0.446 to 0.103]	–	0.066 [-0.206 to 0.339]
B02 (antihemorrhagics)	–	0.037 [-0.073 to 0.147]	0.057	0.087 [-0.019 to 0.193]
B03 (antianemic)	–	-0.068 [-0.220 to 0.084]	–	0.087 [-0.098 to 0.273]
B05 (blood substitutes, perfusion)	-0.174	-0.248 [-0.380 to -0.115]	–	0.022 [-0.109 to 0.153]
B06 (other hematological)	–	–	–	–
C01 (cardiac)	–	0.006 [-0.192 to 0.203]	-0.051	-0.138 [-0.360 to 0.084]
C02 (antihypertensives)	–	0.253 [-0.403 to 0.909]	–	-0.241 [-1.060 to 0.578]
C03 (diuretics)	–	0.140 [-0.332 to 0.612]	–	0.130 [-0.341 to 0.600]
C04 (peripheral vasodilators)	0.275	0.309 [0.041–0.577]	–	-0.239 [-0.648 to 0.170]
C05 (vasoprotectives)	0.001	0.037 [-0.095 to 0.170]	–	0.033 [-0.111 to 0.177]
C07 (beta blocking agents)	-0.094	-0.249 [-0.683 to 0.186]	–	0.029 [-0.341 to 0.400]
C08 (calcium channel blockers)	-0.074	-0.160 [-0.443 to 0.123]	–	-0.157 [-0.413 to 0.099]
C09 (renin-angiotensin system)	-0.219	-0.322 [-0.620 to -0.023]	–	-0.172 [-0.423 to 0.080]
C10 (lipid modifying)	-0.243	-0.344 [-0.579 to -0.109]	–	0.086 [-0.131 to 0.303]
D01 (antifungals)	-0.058	-0.100 [-0.280 to 0.080]	–	0.063 [-0.121 to 0.247]
D02 (emollients, protectives)	–	0.030 [-0.136 to 0.196]	–	-0.110 [-0.291 to 0.072]
D03 (wounds and ulcers)	–	0.053 [-0.260 to 0.365]	–	0.123 [-0.210 to 0.457]
D04 (antipruritics)	-0.126	-0.234 [-0.529 to 0.061]	0.011	0.148 [-0.139 to 0.436]
D05 (antipsoriatics)	-0.202	-0.514 [-1.270 to 0.242]	–	0.022 [-0.515 to 0.559]
D06 (dermatological antibiotics)	0.104	0.148 [-0.002 to 0.298]	–	-0.021 [-0.197 to 0.155]
D07 (dermatological steroids)	-0.002	-0.123 [-0.257 to 0.011]	–	0.005 [-0.138 to 0.148]
D08 (antiseptics, disinfectants)	-0.104	-0.173 [-0.428 to 0.081]	–	0.034 [-0.238 to 0.306]
D09 (medicated dressings)	-0.162	–	–	–
D10 (antiacne)	0.077	0.082 [-0.096 to 0.259]	-0.051	-0.213 [-0.427 to 0.001]
D11 (other dermatological)	–	-0.002 [-0.223 to 0.220]	–	0.065 [-0.169 to 0.299]
G01 (gynecological antiinfectives)	0.120	0.195 [0.019–0.372]	-0.028	-0.260 [-0.536 to 0.016]
G02 (other gynecological)	0.145	0.219 [-0.058 to 0.495]	–	0.075 [-0.294 to 0.444]
G03 (sex hormones)	0.366	0.305 [0.174–0.436]	–	0.032 [-0.202 to 0.267]
G04 (urologicals)	-0.006	-0.127 [-0.517 to 0.262]	0.028	0.209 [-0.142 to 0.560]
H01 (pituitary, hypothalamic)	-0.862	-1.004 [-1.464 to -0.544]	0.209	0.343 [-0.084 to 0.771]
H02 (systemic steroids)	0.071	0.125 [0.000–0.249]	–	-0.051 [-0.174 to 0.071]
H03 (thyroid)	–	-0.063 [-0.340 to 0.214]	–	-0.017 [-0.413 to 0.379]
H04 (pancreatic)	–	-0.083 [-0.917 to 0.752]	–	0.220 [-0.499 to 0.939]
H05 (calcium homeostasis)	–	0.187 [-1.323 to 1.696]	–	-0.090 [-2.152 to 1.972]

– : omitted variable

eTable 8 (continued). Coefficients of the lasso and conventional logistic regression models for prescription of Kampo formulations (tokishakuyakusan, kakkontokasenkyushin'i)

Variable	Tokishakuyakusan		Kakkontokasenkyushin'i	
	Lasso	Conventional logistic [95% confidence interval]	Lasso	Conventional logistic [95% confidence interval]
Prescription, Anatomical Therapeutic Chemical Classification System code				
J01 (antibacterials)	–	-0.036 [-0.153 to 0.081]	0.178	0.236 [0.111–0.360]
J02 (antimycotics)	–	0.270 [-0.640 to 1.180]	–	0.270 [-0.631 to 1.170]
J04 (antimycobacterials)	-0.114	–	–	0.275 [-1.712 to 2.261]
J05 (antivirals)	0.017	0.112 [-0.021 to 0.245]	-0.001	-0.056 [-0.190 to 0.077]
J06 (sera, immunoglobulins)	–	-0.246 [-1.248 to 0.756]	–	-0.158 [-1.049 to 0.734]
J07 (vaccines)	–	-0.010 [-1.185 to 1.165]	–	-0.867 [-2.842 to 1.108]
L01 (antineoplastic)	–	-0.036 [-0.697 to 0.625]	–	0.196 [-0.524 to 0.916]
L02 (endocrine)	–	-0.020 [-0.381 to 0.341]	-0.159	-0.553 [-1.326 to 0.219]
L03 (immunostimulants)	-0.089	-0.406 [-2.076 to 1.264]	–	-0.309 [-2.566 to 1.948]
L04 (immunosuppressants)	-0.310	-0.548 [-1.161 to 0.065]	0.174	0.549 [0.032–1.066]
M01 (antiinflammatory, antirheumatic)	-0.031	-0.073 [-0.183 to 0.038]	-0.029	-0.145 [-0.255 to -0.035]
M02 (joint/muscular topical)	-0.052	-0.123 [-0.260 to 0.015]	0.066	0.150 [0.005–0.295]
M03 (muscle relaxants)	-0.037	-0.088 [-0.283 to 0.107]	–	-0.007 [-0.212 to 0.197]
M04 (antigout)	–	-0.457 [-1.179 to 0.266]	–	-0.039 [-0.342 to 0.263]
M05 (bone)	-0.837	-1.224 [-2.230 to -0.218]	–	-0.212 [-1.053 to 0.628]
M09 (other musculo-skeletal)	–	0.011 [-0.416 to 0.438]	–	0.037 [-0.434 to 0.508]
N01 (anesthetics)	–	-0.007 [-0.169 to 0.156]	-0.018	-0.047 [-0.216 to 0.122]
N02 (analgesics)	-0.115	-0.161 [-0.269 to -0.053]	-0.095	-0.192 [-0.298 to -0.086]
N03 (antiepileptics)	–	-0.010 [-0.229 to 0.208]	–	0.017 [-0.238 to 0.272]
N04 (antiparkinson)	-0.075	-0.217 [-0.634 to 0.199]	-0.051	-0.410 [-1.121 to 0.301]
N05 (psycholeptics)	–	-0.010 [-0.163 to 0.142]	–	-0.031 [-0.205 to 0.143]
N06 (psychoanaleptics)	–	0.040 [-0.174 to 0.254]	-0.129	-0.307 [-0.584 to -0.029]
N07 (other nervous)	0.018	0.047 [-0.175 to 0.269]	-0.137	-0.274 [-0.580 to 0.032]
P01 (antiprotozoals)	–	-0.483 [-2.504 to 1.539]	–	-0.090 [-2.110 to 1.929]
P02 (anthelmintics)	–	–	–	–
P03 (ectoparasiticides)	–	0.005 [-0.230 to 0.241]	-0.023	-0.041 [-0.301 to 0.219]
R01 (nasal)	0.013	0.041 [-0.080 to 0.162]	0.348	0.350 [0.237–0.463]
R02 (throat)	–	0.007 [-0.140 to 0.154]	–	0.008 [-0.129 to 0.146]
R03 (obstructive airway)	–	0.040 [-0.078 to 0.159]	0.143	0.154 [0.045–0.262]
R05 (cough, cold)	-0.041	-0.087 [-0.214 to 0.041]	0.118	0.135 [0.007–0.262]
R06 (antihistamines)	–	0.031 [-0.078 to 0.140]	0.134	0.140 [0.033–0.247]
R07 (other respiratory)	–	-0.020 [-2.073 to 2.034]	–	–
S01 (eye)	0.050	0.052 [-0.083 to 0.187]	0.165	0.219 [0.077–0.362]
S02 (ear)	0.133	0.158 [0.002–0.314]	0.361	0.356 [0.217–0.494]
S03 (eye/ear)	0.058	0.107 [-0.041 to 0.256]	0.052	0.022 [-0.116 to 0.160]
V01 (allergens)	–	-0.026 [-1.032 to 0.980]	–	-0.020 [-0.910 to 0.870]
V03 (other therapeutic)	1.244	1.250 [1.147–1.354]	1.318	1.345 [1.243–1.447]
V04 (diagnostic)	-0.033	-0.074 [-0.304 to 0.157]	-0.014	-0.098 [-0.357 to 0.160]
V06 (nutrients)	–	-0.034 [-0.267 to 0.198]	–	-0.006 [-0.298 to 0.287]
V07 (other non-therapeutic)	–	0.007 [-0.140 to 0.155]	–	0.003 [-0.134 to 0.141]
V08 (contrast media)	–	0.054 [-0.150 to 0.259]	–	0.035 [-0.216 to 0.286]
V09 (diagnostic radiopharmaceuticals)	–	0.134 [-0.523 to 0.790]	–	-0.298 [-1.328 to 0.733]
V10 (therapeutic radiopharmaceuticals)	–	–	–	–
V20 (surgical dressings)	–	–	–	–

– : omitted variable
